# Effects of green tea and roasted green tea on human responses

**DOI:** 10.1038/s41598-024-59383-y

**Published:** 2024-04-13

**Authors:** Chie Kurosaka, Chika Tagata, Sae Nakagawa, Makoto Kobayashi, Shinji Miyake

**Affiliations:** 1 Department of Human, Information and Life Sciences, School of Health Sciences, University of Occupational and Environmental Health, Japan, Kitakyushu, Fukuoka Japan; 2 Central Research Institute, ITOEN, Ltd., Makinohara, Shizuoka Japan; 3 Graduate School of Science and Technology, Chitose Institute of Science and Technology, Chitose, Hokkaido Japan

**Keywords:** Quality of life, Psychology, Biomedical engineering, Occupational health

## Abstract

Our objective was to elucidate the effects of tea consumption on refreshment and stress reduction/recovery through examining the multiple associations among factors such as various physiological responses and task performance. Participants included 20 healthy young men who performed a mental arithmetic task while 11 physiological responses were measured. The experiments were conducted twice under different beverage consumption conditions on separate days. The mental arithmetic task was executed six times in 1 day; participants ingested hot water, green tea, or roasted green tea (hojicha) before each task. Several subjective assessments: subjective fatigue, stress, mental workload, and flow were evaluated after each task. The R–R intervals, heart rate variability spectral components, the Poincaré plot indices (SD1 and SD2) and plethysmogram amplitude tended to decrease during task periods compared to resting periods. Tissue blood volume/flow (TBV, TBF) and near-infrared spectroscopy responses (NIRS) were lower in the tea condition than in the hot water condition. By scrutinizing various indicators, we found that aromatic stimulation of Japanese tea beverages has the potential to induce positive effects, enhance mental task performance, promote refreshment, and alleviate feelings of fatigue. These positive effects were observed even in small quantities and within a short duration, mirroring responses observed in daily consumption.

## Introduction

Japanese tea has become increasingly popular with the rise in health consciousness. Green tea, in particular, is consumed worldwide as a healthy drink because of its characteristic inhibition of cholesterol absorption by the body through tea catechins (gallated bioactive compounds found in green tea), as well as its antioxidant and cavity-preventing (antibacterial) effects^1^. Various types of green tea (sencha, roasted green tea (hojicha), gyokuro, and bancha) currently exist, differing based on the production method. Sencha is the most popular green tea. Sencha is tea leaves grown without blocking sunlight and is produced using a method that involves rolling and drying the tea leaves in several stages. Gyokuro is produced from tea leaves grown with blocking sunlight, therefore it is rich in theanine and poor in catechins. Bancha is mainly produced from tea leaves harvested after summer and less expensive than Sencha. Rosted green tea (hojicha) is a tea made by roasting sencha or bancha at 160–180 °C and has a unique aroma and light astringency. In a study of elderly people in Japan, higher green tea consumption has been reported to lower mortality^2^ and reduce the risk of developing diabetes^3^, colorectal cancer and gastric cancer^2,4,5^. Hursel et al. investigated how catechin- and caffeine-rich teas (CCRTs), such as green tea and oolong tea, maintained and promoted energy expenditure and increased fat oxidation^6^. They suggest that CCRTs’ thermogenic effects may have significant effects on metabolism, fat absorption, and energy intake. Furthermore, green tea was found to lower blood pressure^7^; people who drank green tea for 10 years had smaller waists and lower body fat levels^8^, suggesting that green tea may prevent lifestyle-related diseases.

Green tea contains not only catechins and caffeine but also theanine and vitamins. Catechins have thermogenic and body fat-reducing effects^9^, and long-term continuous consumption has been shown to reduce body weight^10^. In addition, l-theanine, which is abundant in green tea, is known to suppress caffeine-induced excitation^11^. In a study using mice, theanine intake was shown to reduce brain atrophy, learning disabilities, and depressive-like behaviors, as well as stress^12^. Another study showed that l-theanine orally ingested by female university students produced alpha waves in the occipital and parietal regions of the brain, resulting in a possible relaxation effect^13^. Relatedly, a study on the effects of l-theanine at more realistic dietary levels revealed that alpha wave increases were induced in the l-theanine condition regardless of gender^14^.

The aroma of green tea has been further reported to induce positive emotions, increase vitality scores and task performance after stress, and induce positive physiological responses such as electroencephalogram (EEG) activity and salivary chromogranin A (CgA)^15,16^. An odor administration study using two types of Japanese tea with different manufacturing processes, which evaluated electroencephalogram, subjective assessment, and task performance revealed that smelling Japanese tea induced an increase in the band power of alpha and beta waves and enhanced task performance and relaxation scores^15^. In a related experiment, participants consumed warm water and two types of green tea, and the result showed that green tea suppressed the increase in salivary CgA concentration and improved mood after mental workload, indicating that the aroma of green tea could be involved in this effect^16^. Another study investigated autonomic nervous system activity (heart rate, blood pressure, and electrodermal activity) and endocrine salivary cortisol responses to the inhalation of linalool, a component of green tea, demonstrating that this aroma component regulates salivary cortisol levels^17^. The authors examined the results of autonomic activity and concluded that chirality was related to the physiological effects of aromatic substances and that aromatic substances might have different effects on certain physiological indices. Compared with green tea and water, hojicha has been shown to enhance feelings of refreshment and motivation and to peak earlier in the EEG P300 wave (a measure of concentration), suggesting a positive correlation with improved concentration and cognitive ability^18^. Although roasted green tea contains less catechin, theanine, and vitamins than non-roasted green tea, roasting reduces the amount of caffeine, bitterness, and astringency compared to non-roasted green tea, making it easier for pregnant women and children to drink it regardless of the time of day. In addition, hojicha is rich in pyrazine, a component that lends a distinctive aroma and is expected to improve blood circulation, reduce sensitivity to cold, relieve fatigue, and relax the body.

Green tea is ingested in various daily situations such as eating, quenching thirst, alleviating fatigue, want to change mood. Several studies have been conducted on the relationship between the constituents of Japanese tea and disease prevention and health maintenance, and on the psychological effects of aromatic constituents. However, most of these studies are long-term observations or examinations of the effects of consuming large amounts of specific ingredients on the body; few studies have objectively evaluated the physiological responses and subjective amounts of tea consumed in daily life. Furthermore, reports of green tea on multiple physiological responses, task performance, and subjective evaluations are scarce. A variety of elements, such as caffeine, theanine, aroma, taste, are likely to contribute to these effects. Investigating the responses regarding the two types of Japanese tea (green tea and roasted green tea), each with different taste, aroma, and ratios of caffeine and theanine components, would clarify their effects. Therefore, this study measured multiple physiological responses, task performance, and subjective evaluations of the taste and aroma of water, green tea, and roasted green tea (hojicha), examining their interrelationships from various perspectives. By analyzing the relationships among these beverages, we aimed to objectively evaluate the effects of tea consumption on refreshment and stress reduction/recovery. We hypothesize that the consumption of Japanese tea during a mental task will likely have positive effects such as enhancing task performance and alleviating fatigue and stress, and these effects are observed even with the same small amount consumed in daily life.

## Materials and methods

### Participants

Twenty healthy men aged 18–30 years (mean ± SD = 22.8 ± 2.54 years) participated in this study, which eliminated the need to consider female hormonal cycle effects^19,20^. The altered functioning of the autonomic nervous system in the late luteal phase could be associated with diverse psychosomatic and behavioral symptoms appearing premenstrually; specifically, in the premenstrual syndrome group, which is experienced by many women, high frequency component power of heart rate variability and total power were significantly decreased in the late luteal phase compared to the follicular phase^19^. Furthermore, the menstrual cycle has been shown to affect task performance, with a decline in performance observed premenstrually^20^. The sample size was determined based on our previous experiment (not published) and with reference to Cohen’s literature^21^. Participants were excluded from the experiment if they (1) had circulatory problems, such as arrhythmia or were taking medication, (2) smoked, or (3) had dry skin, such as atopic dermatitis. The following conditions were imposed on participants: (1) no heavy drinking and sufficient sleep on the night before the experiment, (2) no strenuous exercise on the day of the experiment, (3) no caffeine consumption until the end of the experiment, and (4) maintain usual daily living conditions.

The experiment started at 9:00 AM or 1:00 PM, and AM and PM were counterbalanced among the participants. All participants were examined twice, under different beverage intake conditions and on different experimental days, with the interval between the first and second experimental days being within one month. All participants provided written informed consent. This study was approved by the Ethics Committee of the University of Occupational and Environmental Health, Japan (Approval No. R2-066), and performed in accordance with relevant guidelines and regulations.

### Procedure

**Figure 1:**
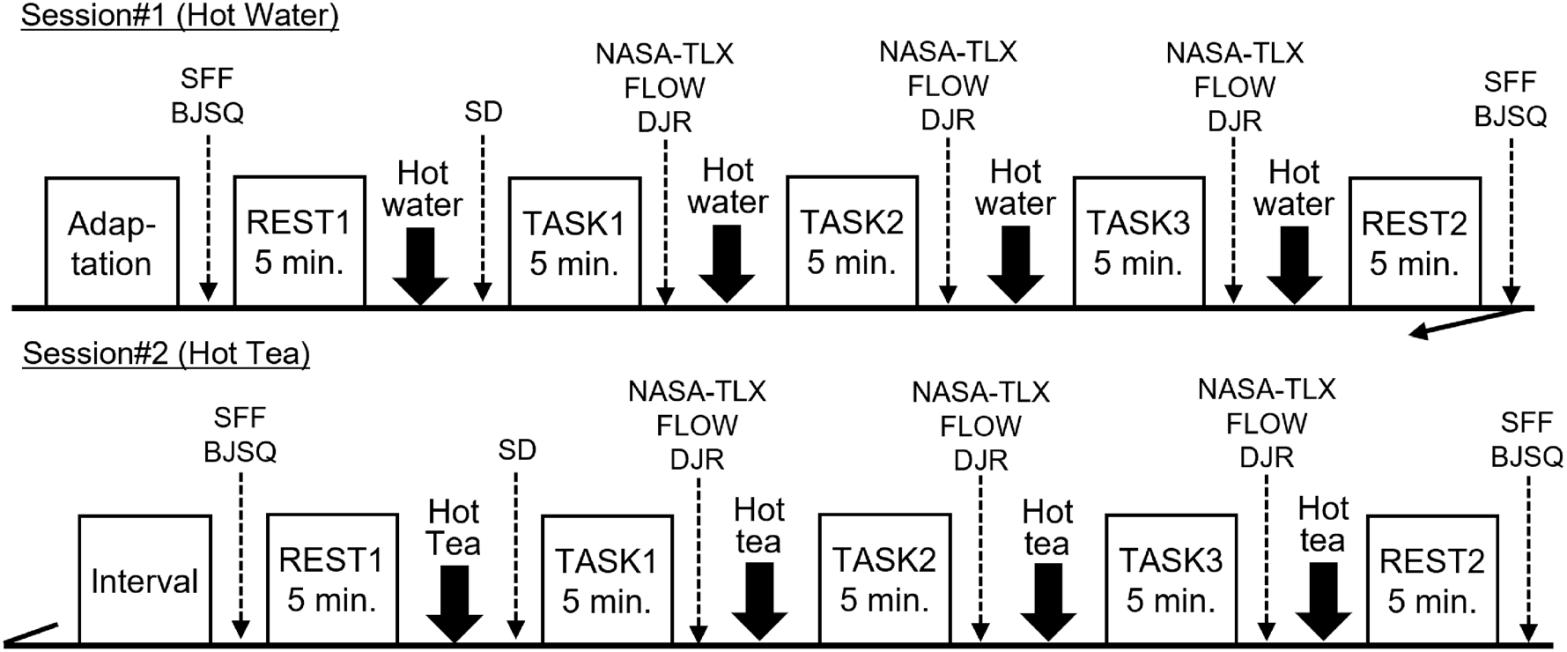
Experimental procedure. *SD* evaluation of drink by semantic differential method, *SFF* subjective fatigue feelings, *BJSQ* brief job stress questionnaire, *NASA-TLX* NASA task load index, *FLOW* flow check list, *DJR* duration judgment ratio.

First, after checking the experimental procedure and instructions for the mental task, participants practiced the mental task. Electrodes and transducers were then attached, and the experiment started after approximately 10 min of acclimation time. The experimental procedure is illustrated in Fig. 1. First, the Subjective Fatigue Feelings (SFF)^22^ questionnaire was administered; subsequently, the subjects were asked to respond to 18 psychological stress reaction items (items #18–35) of the Brief Job Stress Questionnaire (BJSQ; Japan Industrial Safety and Health Association, n.d., Retrieved November 10, 2023) regarding their current state of stress. After these subjective assessments, participants were asked to stay calm (for 5 min), consume a beverage (hot water or green tea), evaluate the beverage in terms of its taste and aroma by semantic differential (SD) method, and perform a mental task (5 min).

The NASA Task Load Index (NASA-TLX)^23,24^, the Flow Experience Checklist (FLOW; 12 questions, simplified version)^25,26^, and the Duration Judgment Ratio (DJR)^27^ tests were then administered. NASA Task Load Index (NASA-TLX) consists of six subscales: Mental demand (MD), Physical demand (PD), Temporal demand (TD), Own Performance (OP), Effort (EF), and Frustration level (FR). All participants were required to answer each subscale using the Visual Analog Scale (VAS) method, ranging from 0 to 100, to indicate low/high levels (own performance scale being poor/good). The FLOW was rated on a 7-point scale (1: strongly disagree, 2: disagree, 3: slightly disagree, 4: undecide, 5: slightly agree, 6: agree, 7: strongly agree) for 12 questions. Regarding the DJR, participants were asked to rate their perception of mental task compared to actual time, 5 min, based on the VAS method (from 0: shortest to 100: longest).

The procedure from beverage intake to DJR was repeated three times; rest recordings (5 min), SFF, and BJSQ were recorded each time. The beverage was evaluated only during the first time, although all participants consumed a beverage four times per each session. After the hot water consumption session, a 10-min break was taken, the beverage type was changed to tea, and the same procedure was repeated. After all experiments were completed, participants reported their daily beverage intake and preferences.

All participants performed the two conditions (hot water + green tea and hot water + roasted green tea) on two separate days. Each condition was provided to the participants in an offsetting order; however, the beverages were administered in the order of hot water, followed by tea. Participants were alone in the sound-proof room and followed instructions from the adjacent monitoring room. Subjective evaluation and mental tasks were displayed on a 27-inch PC display on a desk in front of the participant, and all subjective evaluations and tasks were performed using mouse operation only.

### Procedure for beverage administration

The beverages used included water, green tea, and roasted green tea. The beverages were warmed in a microwave oven, divided into four paper cups (50 ml each), and kept at a temperature of 55 ± 1 °C in a warming cabinet. Temperatures were continuously measured using thermocouples at two locations in the warming cabinet and at the bottom of each paper cup. The results of the beverages’ component analysis by the Japan Food Research Laboratories and Showa Denko Materials Techno-Service Corporation are shown in Supplementary Table 1. Analysis of aroma components, such as 2-Ethyl-3, 5-dimethylpyrazine, tetramethylpyrazine, and 2,3-diethyl-5-methylpyrazine were analyzed by dynamic headspace-gas chromatography (DHS-GC)-/MS using Tenax TA trapping system (GERSTEL GmbH & Co.KG, Germany). The DHS-GC-MS systems used were the GERSTEL MPS autosampler (GERSTEL GmbH & Co.KG), Agilent 7890B GC system (Agilent Technologies, Santa Clara, CA), and Agilent 5977A mass spectrometric degradation system (Agilent). The analytical column was DB-WAX (60 m × 0.25 mm i.d.; d_f_ 0.25 μm, Agilent Technologies).

### Mental task

The mental task is based on the MATH algorithm proposed by Turner et al.^28^. In this task, an addition or subtraction problem and answer are shown on a computer screen. A numerical problem is displayed for 2 s, after which the target number is revealed following the word “EQUALS”.

Participants were required to press the left mouse button if the target number was correct and click the right mouse button if not. The task was performed in machine-paced conditions, and the next problem automatically appeared every 5 s, regardless of whether the participants responded. The task contained five levels of difficulty, as shown in Table 1. However, these formulae differed from those used by Turner et al. The initial question was always at level 3. The next question level increased if the prior answer was correct and decreased otherwise. Participants were instructed to respond with the mouse within 1.5 s of the number being presented and to continue with the next mental arithmetic task even if they could not respond in time. Participants were not informed of their level and were not given feedback on whether their answers were correct.

**Table 1:** Mental arithmetic task level.

Level	Formula	Example
1—easy	2-digit + 1-digit	34 + 7
2	2-digit − 1-digit	78 − 9
3	2-digit ± 2-digit	65 ± 28
4	3-digit + 2-digit	472 + 35
5—difficult	3-digit − 2-digit	597 − 83

### Physiological measurement

The physiological measurement indices and positions are shown in Table 2. All physiological indices were measured continuously throughout the experimental sessions and recorded at a sampling rate of 1 kHz data logger (KEYENCE NR-600/NR2000). To prevent numbness or discomfort during extended noninvasive continuous blood pressure measurements, the blood pressure sensor was temporarily paused after the first half of the session. In the second half of the session, the measurement finger was changed before resuming the blood pressure measurements. Tissue blood volume and blood flow based on the fluctuation of the scattered laser light were measured by using the Laser Doppler Blood Flowmeter. NIRS data were obtained using a two-channel wireless system (Pocket NIRS HM, DynaSence, Hamamatsu, Japan). This device measured changes in levels of oxygenated hemoglobin (oxyhemoglobin–hemoglobin) and deoxygenated hemoglobin (deoxyhemoglobin) at three wavelengths of 735, 810, and 850 nm from the baseline, which is the level when sensors were attached. Concentrations of oxyhemoglobin–hemoglobin and deoxyhemoglobin levels were based on the modified Beer–Lambert law. Data were recorded at a sampling interval of 16 ms and converted to analog data to obtain them simultaneously with other physiological signals at a sampling rate of 1 ms. NIRS sensors were placed on both the left and right sides of the frontopolar (prefront cortex).

**Table 2:** Physiological measurements.

Physiological index	Analysis indices and abbreviation	Measurement site	Measurement instrument model no. (manufacturer)
Electrocardiogram	RRI: R-R interval LF, HF: low/high frequency component SD1, SD2, CVI, CSI: Poincaré Plot indices	CM_5_ lead	SYNAFIT 2200 (NEC SAN’EI)
Respiration	RESP *N/A	Chest	
Electroencephalogram	EEG *N/A	Frontal midline	
Photoelectric plethysmogram	PTGfinger	Left ring finger	MPN2001 (MediSense)
	PTGear	Left earlobe	PPG100C (BIOPAC)
Skin potential level	SPL	Right palm	EDA100C (BIOPAC)
Continuous blood pressure	SBP, DBP, MBP: systolic/diastolic/mean blood pressure	Left index and middle fingers	CNAP MONITOR 500HD (Cnsystems)
Cardiac output	CO		
Tissue blood volume	TBV	Nose tip	FLO-C1 (OMEGA WAVE)
Tissue blood flow	TBF		
Near infra-red spectroscopy	OxyHb/deOxyHb: (de)oxygen hemoglobin totalHb: total hemoglobin	Left and right sides of the forehead	PocketNIRS Duo (DynaSense)

*N/A (not applicable) means excluded from this manuscript for future work.

### Analysis methods

#### Task performance and subjective assessments

The response rate (percentage of questions answered within the time limit) and correct response rate (number of correct answers/total number of questions) for each task, as well as the average level of difficulty, were calculated for task performance. For NASA-TLX, the mean of the adaptive weighting workload (AWWL) and six subscales were calculated; for the FLOW score, the mean of all items and four subscales (AIM, TIME, CHALLENGE, and FEEDBACK) were determined; and for the DJR, the mean of each trial score was obtained. The weighted mean of six subscales of NASA-TLX was calculated without the paired comparisons. The weight coefficient for each subscale was decided by the subscale value itself. For example, the weight coefficients of the maximum and minimum subscales are six and one, respectively. The weighted mean of this method is called the Adaptive Weighted Workload (AWWL) score and indicates a high correlation with the original weighted workload (WWL) score^24^.

Task performance and NASA-TLX data were incomplete for three participants, whereas FLOW and DJR data were missing for one participant.

For all indices except FLOW, standardized scores were calculated for six blocks (3 task blocks × 2 sessions) for each participant per condition; since the FLOW score is an index with meaning in absolute values, we used the rating value. Multiple comparisons were conducted for all measures using the repeated two-factor analyses of variance and Ryan–Einot–Gabriel–Welsch F test (R–E–G–W F test) for the two drinking sessions and three task blocks. Regarding the SFF score, five factors (feeling of drowsiness: Factor I; feeling of instability: Factor II; feeling of uneasiness: Factor III; feeling of local pain or dullness: Factor IV; and feeling of eyestrain: Factor V) were calculated. The SFF total score was derived from the sum of these five factors. The BJSQ and SFF scores were compared before and after each session by separately calculating the standardized scores of the four blocks for each participant (paired t-test). Cohen’s d was applied for effect sizes^21,29^. A detailed relationship between task performance and subjective evaluation has been reported by Tagata et al.^30^.

#### Physiological signals

The R–R interval (RRI) was obtained from the ECG waveform by detecting the R wave at each beat. We then calculated the mean values of the RRI, heart rate variability (HRV) such as low frequency (LF) component, high frequency (HF) component, LF/HF ratio, and Poincaré plot indices (SD1, SD2, Cardiac Vagal Index: CVI, and Cardiac Sympathetic Index: CSI)^31^. SD2 is the length of the longitudinal axis of the ellipse which is parallel with the line RRI_*k*_ = RRI_(*k*+1)_ and SD1 is the length of the vertical axis to this line. These indices mean the standard deviation of the Poincaré plot perpendicular/parallel to each axis. For continuous blood pressure, mean blood pressure (MBP) was calculated from systolic blood pressure (SBP) and diastolic blood pressure (DBP. MBP = DBP + (SBP-DBP/3)) to obtain the total peripheral resistance (TPR = MBP/CO: CO is cardiac output). Baroreceptor reflex sensitivity (BRS) was simply calculated using the square root of the ratio of LF_RR_ and LF_SBP_ (0.05–0.15 Hz) components in the spectral analysis of RRI and SBP^32^. The PTG amplitudes were obtained by simply calculating them based on the knowledge of a significant correlation between PTG amplitude and the standard deviation of PTG waveform^33^.

For all 24 indicators, the mean value for each block and standardized scores for each participant by condition (by date of administration) were calculated. Repeated two-factor analysis of variance (condition 2 × block 5) and the R–E–G–W F tests were conducted when the main effect was significant. When a significant interaction was found, multiple comparisons were made for all pairs using the Holm method. The significance level was set at *p* < 0.05 and *p* < 0.10 was considered to be marginally significant.

Correlation coefficients among the HRV indices, BRS, and TPR were calculated for each session, and then mean correlation coefficients were obtained by inverse z-transformation of the mean values after Fisher’s z-transformation.

One participant with a large RRI variability (SDNN = 146.6 ms) and a large deviation from the normal heart rate (SDNN = 50 ± 16 ms) was excluded from the HRV analysis (Smirnov-Grubbs test: *p* = 0.006). Two participants had missing blood pressure data owing to incomplete measurements. Therefore, HRV analysis was conducted for 19 participants, and blood pressure data analysis was performed for 18 participants.

### Ethics approval

All participants provided written informed consent. This study was approved by the Ethics Committee of the University of Occupational and Environmental Health, Japan (Approval No. R2-066), and performed in accordance with relevant guidelines and regulations.

## Results

In all trials, participants consumed water in the first half session (session#1) and green tea or roasted green tea in the second half session (session#2). Within each session, 5 min blocks were denoted as REST1 and REST2 to indicate resting periods and TASK1 to TASK3 to indicate mental tasks.

### Task performance and subjective assessments

**Figure 2:**
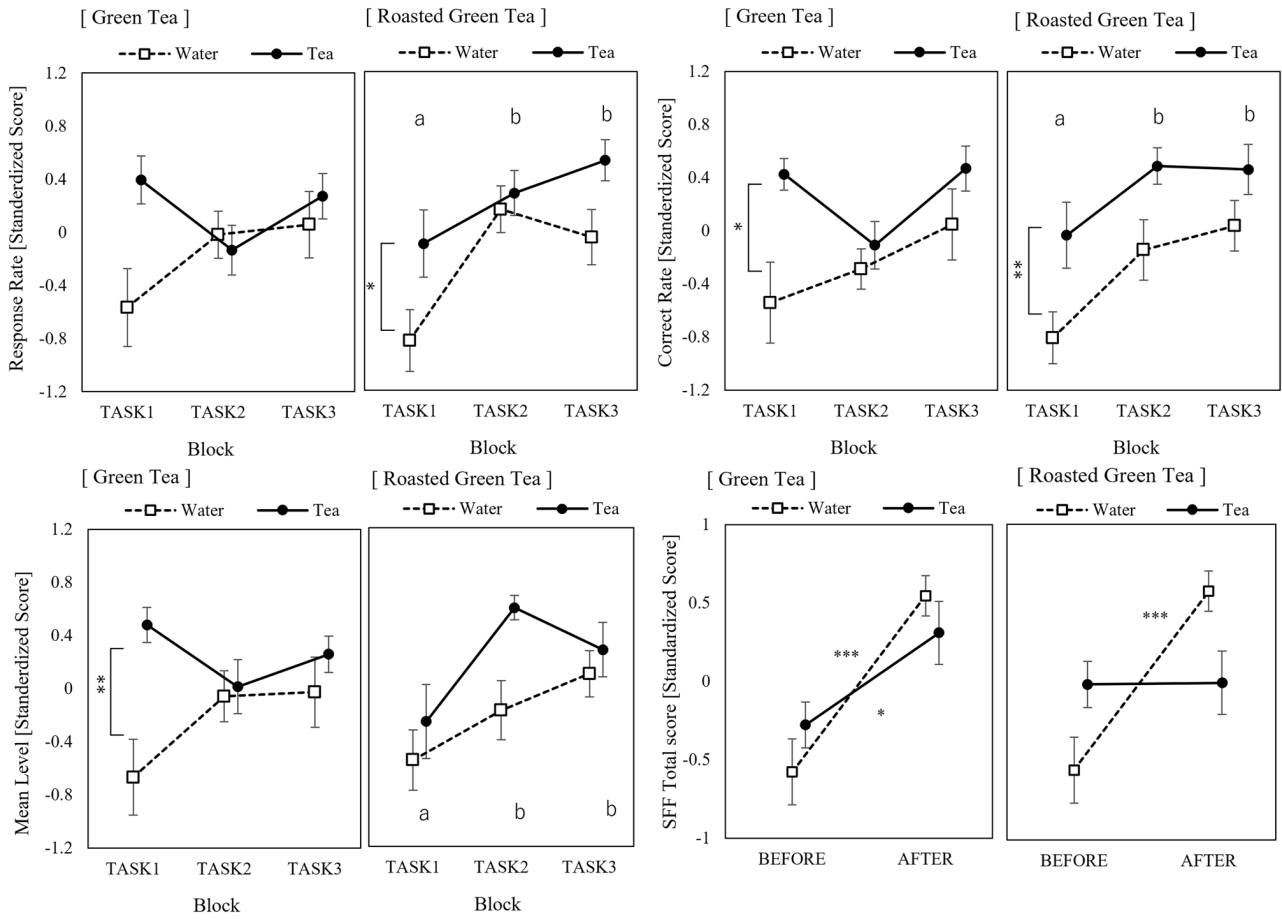
Task performance and SFF total score. Bars indicate standard errors of the mean. The alphabets indicate homogenous subset results based on the REGW F-test.

In the green tea condition, the average problem level was significantly higher in the tea session than in the water session (*F*(1, 16) = 9.95, *p* = 0.006, 1 − *β* = 0.84, *partial η*^2^ = 0.38), but no significant trend was observed in the other indicators. However, in the roasted green tea condition, the correct response rate and the response rate were significantly higher in the tea session than in the water session (response rate: *F*(1, 16) = 4.55, *p* = 0.049, 1 − *β* = 0.52, *partial η*^2^ = 0.22; correct response rate: *F*(1, 16) = 8.99, *p* = 0.008, 1 − *β* = 0.80, *partial η*^2^ = 0.36). These task performance indicators also showed main effects between blocks, with significantly higher values in TASK2 and TASK3 compared to TASK1 (response rate: *F*(2, 32) = 8.93, *p* = 0.001, 1 − *β* = 0.95, *partial η*^2^ = 0.36; correct response rate: *F* (2, 32) = 5.41, *p* = 0.011, 1 − *β* = 0.78, *partial η*^2^ = 0.25). Across sessions, task performance was higher in the tea condition than in the hot water condition, and across blocks, task performance was better in TASK2 and TASK3 than in TASK1 in the roasted green tea condition (Fig. 2).

The results in NASA-TLX, FLOW, and DJR showed no obvious trends. Supplementary Table 2 shows the analysis of variance results for all measures. There were no significant differences in BJSQ scores between sessions, and the SFF total score increased significantly after the task in all conditions except the second half session of the roasted green tea condition (Fig. 2). session#1 of the green tea: *p* < 0.001, 95% confidence intervals (CI) 0.53–1.60. session#2 of the green tea-tea: *p* = 0.036. session#1 of the roasted green tea: *p* = 0.001, 95% CI 0.54–1.72). For each item of the SFF, feelings of uneasiness (Factor III) and local pain or dullness (Factor IV) showed a similar trend to the SFF total score; these scores did not increase after the tasks only in the roasted green tea condition. The feeling of drowsiness (Factor I) showed an increasing trend after the task only in the W condition (session#1 of the green tea: *p* = 0.002, 95% CI 0.26–1.46. session#1 of the roasted green tea: *p* = 0.077, 95% CI − 0.10 to 1.19).

### Physiological signals

**Figure 3:**
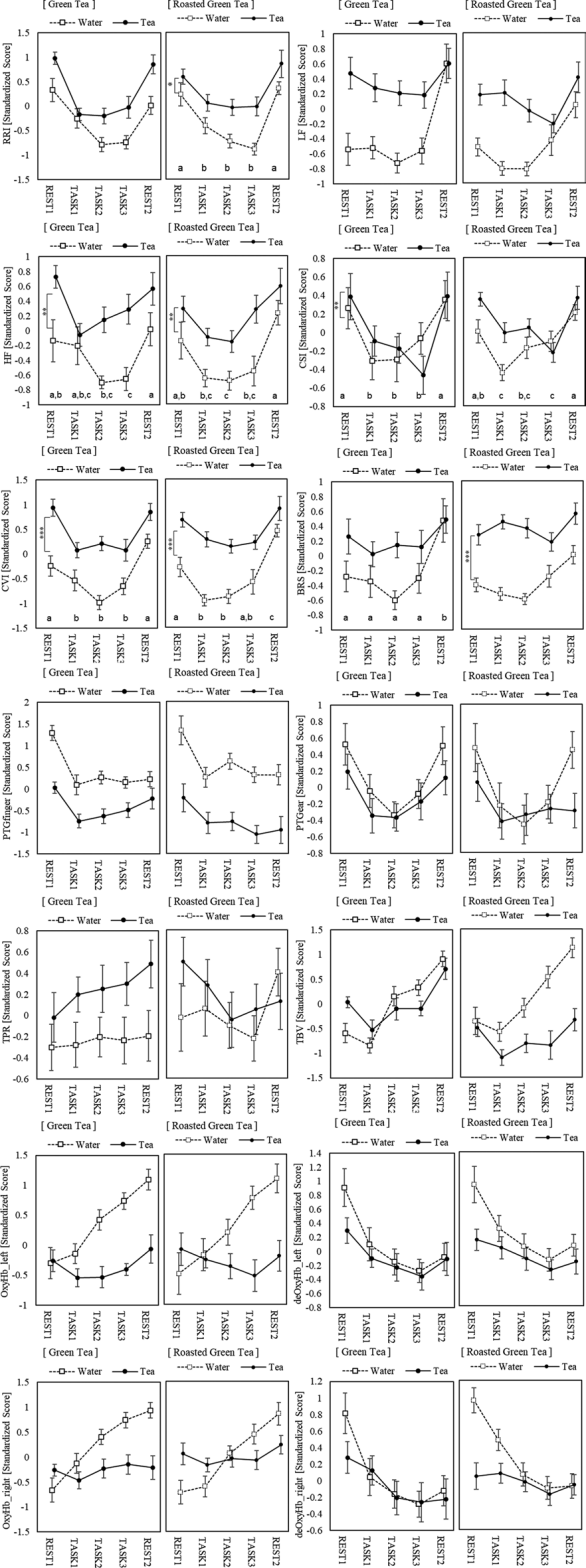
Physiological responses. Bars indicate standard errors of the mean. The alphabets (**a–d**) indicate homogenous subset results based on the REGW F-test. Asterisks indicate the significant differences between sessions (***p < 0.001, **p < 0.01).

The physiological responses under each condition are presented in Table 3. The physiological responses that exhibited distinct trends in each session are shown in Fig. 3. The statistical analysis results for all physiological responses are provided in Supplementary Table 3.

**Table 3:** The statistical values (mean ± SEM) for each physiological response under each condition.

	Session#1 (water)					Session#2 (tea)				
	REST1	TASK1	TASK2	TASK3	REST2	REST1	TASK1	TASK2	TASK3	REST2
(1) Water and green tea condition										
RRI [ms]	887.3 ± 24.5	865.1 ± 21.5	847.9 ± 20.1	848.7 ± 19.3	885.5 ± 22.5	915.1 ± 20.2	872.0 ± 17.4	874.1 ± 16.8	882.1 ± 18.1	914.2 ± 19.3
LF [ms^2^]	611.9 ± 164.7	427.6 ± 73.3	400.5 ± 59.6	438.1 ± 68.1	985.6 ± 211.9	909.7 ± 198.4	776.8 ± 151.1	737.6 ± 155.0	704.9 ± 139.7	987.3 ± 204.3
HF [ms^2^]	477.4 ± 125.8	375.6 ± 80.4	354.0 ± 81.2	340.9 ± 73.7	464.3 ± 88.7	604.8 ± 126.3	423.1 ± 72.0	474.6 ± 92.2	538.6 ± 120.6	584.6 ± 120.2
ln LF/HF	0.61 ± 0.14	0.63 ± 0.15	0.48 ± 0.12	0.69 ± 0.15	0.76 ± 0.12	0.58 ± 0.11	0.73 ± 0.16	0.72 ± 0.14	0.60 ± 0.14	0.69 ± 0.13
SD1 [ms]	25.0 ± 0.0	23.6 ± 0.0	22.6 ± 0.0	22.7 ± 0.0	26.9 ± 0.0	30.3 ± 0.0	27.1 ± 0.0	28.2 ± 0.0	29.1 ± 0.0	30.8 ± 0.0
SD2 [ms]	79.1 ± 7.1	66.9 ± 5.3	64.3 ± 5.1	66.9 ± 4.0	89.6 ± 7.4	102.2 ± 8.5	80.1 ± 6.2	82.0 ± 6.7	77.8 ± 6.3	103.8 ± 9.7
CSI [a.u.]	3.48 ± 0.27	2.96 ± 0.17	3.01 ± 0.22	3.19 ± 0.23	3.51 ± 0.22	3.64 ± 0.29	3.16 ± 0.23	3.14 ± 0.24	2.85 ± 0.16	3.55 ± 0.29
CVI [a.u.]	3.21 ± 0.08	3.15 ± 0.06	3.11 ± 0.07	3.14 ± 0.06	3.32 ± 0.07	3.43 ± 0.07	3.28 ± 0.07	3.31 ± 0.07	3.29 ± 0.07	3.43 ± 0.08
SBP [mmHg]	119.7 ± 3.8	124.4 ± 4.4	125.2 ± 3.8	127.4 ± 3.8	126.4 ± 3.6	117.1 ± 3.3	123.8 ± 3.6	127.0 ± 4.9	126.8 ± 3.9	127.1 ± 4.1
DBP [mmHg]	74.8 ± 3.5	78.5 ± 4.9	79.7 ± 4.3	78.5 ± 3.9	76.8 ± 3.3	72.7 ± 3.8	77.4 ± 3.9	79.1 ± 4.6	78.7 ± 4.2	78.6 ± 4.7
MBP [mmHg]	89.7 ± 3.3	93.8 ± 4.5	94.9 ± 3.9	94.8 ± 3.7	93.3 ± 3.1	87.5 ± 3.4	92.9 ± 3.6	95.1 ± 4.4	94.8 ± 3.9	94.7 ± 4.3
BRS [mmHg/ms]	10.28 ± 1.29	9.28 ± 0.87	9.21 ± 0.98	9.71 ± 1.08	13.89 ± 2.09	11.24 ± 0.95	10.67 ± 0.87	10.89 ± 0.85	10.81 ± 0.87	12.61 ± 1.31
CO [l/min]	6.18 ± 0.23	6.37 ± 0.22	6.48 ± 0.25	6.50 ± 0.27	6.33 ± 0.23	5.71 ± 0.24	5.86 ± 0.24	5.93 ± 0.25	5.94 ± 0.25	5.84 ± 0.24
TPR [a.u.]	14.9 ± 0.8	15.1 ± 1.0	15.2 ± 0.9	15.2 ± 1.0	15.2 ± 0.8	15.9 ± 1.0	16.4 ± 1.0	16.6 ± 1.2	16.6 ± 1.1	17.0 ± 1.3
PTGfinger [a.u.]	0.200 ± 0.020	0.168 ± 0.019	0.177 ± 0.021	0.170 ± 0.018	0.172 ± 0.018	0.165 ± 0.017	0.141 ± 0.016	0.147 ± 0.017	0.154 ± 0.020	0.160 ± 0.019
PTGear [a.u.]	0.210 ± 0.025	0.185 ± 0.026	0.175 ± 0.024	0.179 ± 0.024	0.199 ± 0.024	0.201 ± 0.025	0.167 ± 0.021	0.174 ± 0.024	0.180 ± 0.027	0.184 ± 0.024
TBV [a.u.]	0.665 ± 0.028	0.654 ± 0.030	0.696 ± 0.034	0.703 ± 0.035	0.727 ± 0.034	0.690 ± 0.035	0.659 ± 0.034	0.684 ± 0.038	0.683 ± 0.036	0.712 ± 0.034
TBF [a.u.]	0.850 ± 0.041	0.813 ± 0.043	0.883 ± 0.051	0.893 ± 0.047	0.950 ± 0.047	0.898 ± 0.051	0.841 ± 0.051	0.875 ± 0.054	0.864 ± 0.050	0.930 ± 0.055
SPL [mV]	− 57.0 ± 5.5	− 49.1 ± 5.1	− 52.7 ± 5.0	− 50.4 ± 4.7	− 56.6 ± 5.1	− 62.8 ± 4.9	− 58.0 ± 5.0	− 56.9 ± 5.2	− 56.3 ± 5.7	− 58.7 ± 6.1
deOxyHb_left [a.u.]	0.417 ± 0.096	0.298 ± 0.092	0.322 ± 0.095	0.315 ± 0.101	0.386 ± 0.110	0.380 ± 0.126	0.345 ± 0.131	0.383 ± 0.144	0.417 ± 0.136	0.368 ± 0.128
OxyHb_left [a.u.]	0.402 ± 0.094	0.342 ± 0.098	0.367 ± 0.082	0.413 ± 0.089	0.709 ± 0.144	0.609 ± 0.166	0.493 ± 0.144	0.572 ± 0.146	0.517 ± 0.142	0.896 ± 0.182
totalHb_left [a.u.]	0.540 ± 0.108	0.446 ± 0.121	0.534 ± 0.124	0.572 ± 0.128	0.634 ± 0.153	0.600 ± 0.148	0.496 ± 0.162	0.559 ± 0.163	0.600 ± 0.154	0.754 ± 0.181
deOxyHb_right [a.u.]	0.453 ± 0.111	0.415 ± 0.125	0.408 ± 0.137	0.466 ± 0.140	0.404 ± 0.132	0.475 ± 0.126	0.498 ± 0.136	0.599 ± 0.146	0.618 ± 0.155	0.493 ± 0.129
OxyHb_right [a.u.]	0.506 ± 0.102	0.571 ± 0.109	0.694 ± 0.118	0.714 ± 0.120	0.782 ± 0.116	0.543 ± 0.131	0.639 ± 0.132	0.647 ± 0.137	0.680 ± 0.139	0.604 ± 0.131
totalHb_right [a.u.]	0.422 ± 0.088	0.482 ± 0.097	0.591 ± 0.105	0.648 ± 0.116	0.736 ± 0.112	0.575 ± 0.139	0.626 ± 0.140	0.680 ± 0.144	0.646 ± 0.150	0.626 ± 0.144
(2) Water and roasted green tea condition										
RRI [ms]	864.5 ± 29.8	842.0 ± 27.3	830.8 ± 28.5	825.6 ± 26.3	872.0 ± 28.1	881.9 ± 27.9	859.9 ± 23.8	858.4 ± 25.2	860.8 ± 27.9	895.5 ± 31.2
LF [ms^2^]	651.7 ± 242.9	384.1 ± 95.5	364.9 ± 65.6	500.7 ± 111.9	805.8 ± 186.5	945.0 ± 306.5	893.6 ± 229.3	697.0 ± 135.7	735.2 ± 181.9	1317.4 ± 460.1
HF [ms^2^]	808.5 ± 374.5	373.4 ± 107.7	333.0 ± 87.3	563.8 ± 266.1	562.2 ± 120.4	759.4 ± 259.6	468.0 ± 111.8	412.8 ± 92.8	585.3 ± 146.7	960.1 ± 406.4
ln LF/HF	0.85 ± 0.18	0.62 ± 0.15	0.88 ± 0.17	0.66 ± 0.17	0.60 ± 0.12	0.72 ± 0.16	0.96 ± 0.16	0.76 ± 0.16	0.65 ± 0.17	0.68 ± 0.15
SD1 [ms]	26.0 ± 0.0	22.6 ± 0.0	21.8 ± 0.0	26.3 ± 0.0	27.0 ± 0.0	29.1 ± 0.0	26.9 ± 0.0	25.6 ± 0.0	28.4 ± 0.0	32.8 ± 0.0
SD2 [ms]	71.2 ± 7.3	57.1 ± 5.8	62.2 ± 6.4	70.1 ± 7.2	86.6 ± 8.0	96.3 ± 8.8	83.8 ± 9.4	80.6 ± 7.1	77.3 ± 7.9	104.1 ± 9.7
CSI [a.u.]	3.47 ± 0.35	2.74 ± 0.18	3.18 ± 0.33	3.38 ± 0.32	3.59 ± 0.27	3.80 ± 0.32	3.24 ± 0.19	3.35 ± 0.22	3.09 ± 0.29	3.83 ± 0.34
CVI [a.u.]	3.13 ± 0.10	3.02 ± 0.09	3.03 ± 0.09	3.12 ± 0.10	3.28 ± 0.09	3.36 ± 0.08	3.26 ± 0.09	3.24 ± 0.08	3.25 ± 0.09	3.41 ± 0.10
SBP [mmHg]	117.8 ± 4.3	123.8 ± 4.9	126.9 ± 5.0	125.5 ± 5.4	127.0 ± 5.5	119.5 ± 3.9	119.3 ± 3.6	119.7 ± 4.1	122.5 ± 4.7	123.3 ± 4.6
DBP [mmHg]	73.5 ± 5.5	75.2 ± 3.0	74.6 ± 3.3	75.1 ± 3.2	78.2 ± 4.1	70.9 ± 2.4	74.4 ± 4.1	71.3 ± 3.6	73.5 ± 3.6	72.8 ± 3.3
MBP [mmHg]	88.3 ± 4.9	91.4 ± 3.2	92.0 ± 3.5	91.9 ± 3.7	94.5 ± 4.2	87.1 ± 2.6	89.4 ± 3.6	87.4 ± 3.6	89.9 ± 3.8	89.7 ± 3.6
BRS [mmHg/ms]	7.99 ± 1.29	7.03 ± 0.83	6.87 ± 0.74	8.14 ± 1.04	9.57 ± 1.20	11.04 ± 1.18	11.30 ± 0.96	11.15 ± 1.01	10.83 ± 1.30	13.01 ± 1.86
CO [l/min]	6.36 ± 0.20	6.51 ± 0.20	6.68 ± 0.21	6.61 ± 0.19	6.32 ± 0.21	5.96 ± 0.21	6.23 ± 0.24	6.30 ± 0.25	6.34 ± 0.24	6.25 ± 0.25
TPR [a.u.]	14.1 ± 0.9	14.3 ± 0.7	14.0 ± 0.7	14.1 ± 0.7	15.3 ± 1.0	14.9 ± 0.7	14.8 ± 0.9	14.4 ± 0.9	14.7 ± 0.9	14.9 ± 0.9
PTGfinger [a.u.]	0.205 ± 0.021	0.181 ± 0.020	0.190 ± 0.021	0.181 ± 0.018	0.181 ± 0.018	0.164 ± 0.013	0.152 ± 0.016	0.153 ± 0.017	0.144 ± 0.015	0.148 ± 0.013
PTGear [a.u.]	0.227 ± 0.035	0.204 ± 0.037	0.195 ± 0.032	0.196 ± 0.029	0.205 ± 0.023	0.195 ± 0.021	0.176 ± 0.016	0.184 ± 0.021	0.186 ± 0.023	0.187 ± 0.023
TBV [a.u.]	0.700 ± 0.024	0.688 ± 0.026	0.706 ± 0.028	0.730 ± 0.032	0.758 ± 0.029	0.685 ± 0.030	0.658 ± 0.025	0.665 ± 0.027	0.662 ± 0.032	0.681 ± 0.029
TBF [a.u.]	0.901 ± 0.046	0.883 ± 0.051	0.910 ± 0.052	0.954 ± 0.057	1.005 ± 0.056	0.886 ± 0.055	0.851 ± 0.052	0.856 ± 0.055	0.844 ± 0.061	0.877 ± 0.063
SPL [mV]	− 65.7 ± 8.1	− 56.7 ± 8.4	− 57.9 ± 8.3	− 56.3 ± 8.9	− 62.3 ± 9.0	− 66.7 ± 8.4	− 63.6 ± 8.3	− 56.7 ± 7.5	− 54.8 ± 6.3	− 53.1 ± 7.3
deOxyHb_left [a.u.]	0.532 ± 0.146	0.470 ± 0.135	0.403 ± 0.134	0.436 ± 0.143	0.508 ± 0.152	0.702 ± 0.171	0.546 ± 0.169	0.603 ± 0.181	0.608 ± 0.185	0.585 ± 0.185
OxyHb_left [a.u.]	0.404 ± 0.138	0.572 ± 0.164	0.578 ± 0.165	0.628 ± 0.177	0.588 ± 0.169	0.608 ± 0.188	0.555 ± 0.195	0.555 ± 0.196	0.598 ± 0.203	0.633 ± 0.210
totalHb_left [a.u.]	0.648 ± 0.192	0.580 ± 0.223	0.595 ± 0.230	0.638 ± 0.248	0.657 ± 0.235	0.718 ± 0.263	0.742 ± 0.278	0.700 ± 0.283	0.761 ± 0.282	0.887 ± 0.290
deOxyHb_right [a.u.]	0.422 ± 0.094	0.368 ± 0.096	0.358 ± 0.104	0.415 ± 0.126	0.447 ± 0.131	0.444 ± 0.126	0.327 ± 0.126	0.379 ± 0.131	0.414 ± 0.134	0.393 ± 0.132
OxyHb_right [a.u.]	0.365 ± 0.117	0.393 ± 0.128	0.481 ± 0.136	0.590 ± 0.149	0.657 ± 0.147	0.495 ± 0.145	0.429 ± 0.142	0.468 ± 0.151	0.512 ± 0.155	0.551 ± 0.157
totalHb_right [a.u.]	0.361 ± 0.120	0.391 ± 0.128	0.421 ± 0.146	0.499 ± 0.157	0.661 ± 0.161	0.490 ± 0.168	0.484 ± 0.184	0.476 ± 0.188	0.509 ± 0.185	0.574 ± 0.188

*RRI* R wave interval, *LF and HF* low/high components of heart rate variability, *SD1, SD2, CSI, and CVI* Poincaré plot indices, *SBP DBP, and MBP* systolic/diastolic/mean blood pressure, *BRS* baroreflex sensitivity, *CO* cardiac output, *TPR* total peripheral vascular resistance, *PTG* plethysmogram on finger/ear, *TBV and TBF* tissue blood volume/flow, *SPL* skin potential level, *(de)OxyHb* oxygen hemoglobin, *[a.u.]* the physiological indices unit, recorded as the transducer output voltage.

In the green tea condition, the RRI, HRV spectral indices, and PTGfinger amplitude showed interaction effects. Analysis of the differences between blocks in each condition showed that, compared with resting periods, all indices tended to decrease during task periods. For the Poincaré plot indices, both SD1 and SD2 showed significant differences in both Session and Block. They were smaller in the hot water condition than in the tea condition and tended to decrease during task periods compared with resting periods. The CVI showed a similar tendency to SD1 and SD2, but the CSI did not show a consistent trend. Blood pressure was significantly higher during the task periods than during the resting periods, and there was no clear trend in TPR, and skin potential level (SPL). The tissue blood volume (TBV), tissue blood flow (TBF), and near infrared spectroscopy (NIRS) responses were significantly lower in the tea condition than in the hot water condition. The EEG results are not included in this study, as the study is specifically on autonomic nervous system activity. The EEG results will be reported separately in a future study.

Table 4 shows the correlation coefficients of the HRV spectral analysis indices, which are reported to sympathetic activity (LF/HF, CSI) and parasympathetic activity (HF, CVI, and BRS) indices. High correlations were observed between parasympathetic indices.

**Table 4:** Correlations between the HRV indices.

	Green tea condition		Roasted green tea condition	
	Water	Tea	Water	Tea
LF/HF and CSI	0.631**	0.247	0.682***	0.631**
HF and CVI	0.728***	0.806***	0.878***	0.804***
HF and BRS	0.508*	0.420*	0.747***	0.335^†^
CVI and BRS	0.658**	0.365^†^	0.768***	0.373^†^

Asterisks indicate the significant correlation for each session (***p < 0.001, **p < 0.01, *p < 0.05, ^†^p < 0.10).

## Discussion

Our study results demonstrate that tea intake improved mental task performance, such as the correct response rate of machine-paced mental arithmetic tasks compared to the hot water condition. Particularly in the roasted tea condition, the response rate also improved and the task performance increased with the number of trial repetitions. Considering the ingestion of tea ingredients, it was not possible to counterbalance the order of the hot water and tea conditions in this study. Therefore, fatigue was expected to accumulate in the later tea condition. As shown in Fig. 2, task performance was not stable; a slight sluggishness in the green tea condition was shown in TASK2. However, the SFF results indicated that fatigue did not increase after the task in the roasted green tea condition; moreover, task performance increased with the number of task repetitions. Subjective assessments such as the NASA-TLX and stress scores showed no significant changes in either condition. These results suggest that only the roasted green tea condition might have anti-fatigue properties, even though the mental workload demands and stress levels were consistent across all conditions. It is entirely possible that the level of arousal obtained with drowsiness (SFF factor I) is related to the anti-fatigue effect. One factor that contributes to maintaining wakefulness under tea conditions is the tea components’ arousal effects, specifically caffeine^34^. It is also reported that caffeine (40 mg) in combination with l-theanine (97 mg) helps to improve task performance, subjective arousal level, and self-reported tiredness^35^. The half-life of caffeine in the bloodstream is relatively broad, ranging from 2 to 8 h (average of approximately 4 h), indicating a gradual decline^36^. If the results of this study were influenced by oral caffeine intake, the effects could be expected to persist for an extended period. However, most components, including caffeine and l-theanine, are reduced in roasted green tea compared to green tea. Further, as discussed later, the amount of caffeine consumed in this study was very small compared with that in previous research. Considering the rate of ingredient absorption into the body, the potential impact of oral caffeine intake in this study is considered low. In contrast, the only components that are more prevalent in roasted green tea than in green tea are pyrazines and aromatic components (Supplementary Table 1). Although Murao et al.^15^ have reported that aroma of green tea increases feeling of relaxation and vitality through the odor stimuli experiment, a direct relationship between pyrazines and arousal level or anti-fatigue has not been reported. Our findings could be the first report of such a relationship, and further studies on the aroma components of tea should be conducted in more detail.

RRI is well-known as a physiological index sensitive to mental stress, typically resulting in an increase in heart rate (decrease in RRI) during periods of mental stress^37^. A similar tendency was observed in this study. Although the LF component has been reported to decrease during mental tasks in HRV spectral analysis^38^, the same change was not observed in this study. This might be attributed to effects such as participants’ before-task tension. The LF responses during mental tasks are not consistently uniform. Therefore, both LF and LF/HF ratios using these values may not serve as reliable indicators for assessing mental workload. After the task, the LF component appeared to have recovered to its resting level, and in the REST2 block, comparable levels were observed in hot water and tea conditions. Indeed, the LF component also decreased during the task block in the tea condition; however, the magnitude of this decrease was smaller than that observed in the hot water condition. The familiarity effect on the task may have influenced the LF component results. However, considering that the same task was repeated three times in the hot water condition, and that task performance did not necessarily correlate with the number of task repetitions, we could not rule out that the tea might have influenced LF component responses. HF is a component that reflects respiratory sinus arrhythmia (RSA) and is known to be eliminated by vagal blockade^39,40^. There were significant differences between sessions in the HF component; thus, it is possible that tea ingredients affect vagal activity. However, LF/HF ratio did not exhibit a consistent trend. Although the LF/HF ratio has been reported as an indicator of the balance between—and ratio of—sympathetic and parasympathetic activity^41^, many researchers have questioned this point^38,42–44^. Computation of the autonomic balance as the LF/HF ratio is based on a hypothesis that LF is mainly mediated by the sympathetic nervous system and the physiological assumption of autonomic reciprocity^38^. However, LF reflects baroreflex function, originating from vagal activity^42^. Therefore, LF does not reflect sympathetic nervous system activity^43^. Furthermore, the autonomic reciprocity is not supported by current research and fractional transformation such as LF/HF distorts the data, making the applied indices dubious^38^. Billman^44^ claims that LF/HF cannot accurately quantify sympatho-vagal balance. Therefore, HRV spectral analysis requires careful discussion considering other related physiological responses. Regarding the Poincaré plot indices, both SD1 and SD2 decreased during work because of RRI reduction (heart rate increased), and because the Poincaré plot ellipse became smaller. SD1 and Root Mean Square of Successive Differences (RMSSD) have been reported to be mathematically equivalent HRV indices^45^, and the CVI obtained as the product of SD1 and SD2 has been reported to be associated with parasympathetic nervous system activity^31^. The BRS is a reflex vagal activity that is reduced during mental arithmetic tasks and is related to task performance^46,47^. del Paso et al. propose that the association between mental arithmetic tasks and BRS may be modulated by gender and blood pressure. In this study, BRS was obtained using a noninvasive method proposed by DeBoer et al.^32^. Guzik et al.^48^ suggest that CVI may be related to parasympathetic indices such as HF, RMSSD, and BRS. On the other hand, in a review that investigated the Poincaré plot indices and other indices during resting and mental arithmetic tasks, Allen et al.^49^ showed that CVI is unlikely to produce results similar to those of other indices. Studies conducted on HRV analysis indicate its interest and importance as a measure of autonomic venous system activity, despite that it is not being widely used in clinical practice^48,50^. Therefore, Nunan et al.^50^ identified various factors such as the analysis method, duration of electrocardiogram recording, assessment conditions, and participant characteristics. In our previous experiments, we have concurrently measured these indices and investigated their correlations under standardized measurement conditions and analytical methods. However, these indices rarely showed consistently high correlations. Interestingly, in this study, indices commonly reported as parasympathetic nervous system activity, namely HF, CVI, and BRS, showed a high correlation. Nevertheless, as mentioned above, these indices have been subject to considerable debate, and it is difficult to interpret them uniquely. We believe that a multifaceted analysis of data obtained through simultaneous measurement of various indices, as in our study, will deepen this discussion. Future studies should explore composite analyses, including blood pressure, pulse waves, and EDA activity, to further enhance our understanding. In addition, our previous experiments (not published) suggest that fingertip and earlobe pulse waves exhibit different reactivity and the amplitude of the plethysmogram on fingertip may show a characteristic tendency in the recovery process. The difference in responses between two sites in this study appears to support this tendency. The details will be published in future research.

The physiological responses of crucial interest in this study were changes in the TBV, TBF of the nose tip, and NIRS indices. NIRS values were measured as the amount of relative change from the starting point of measurement^51^. These indices showed significant differences between the blocks in the hot water condition, but there were few changes in the tea condition. Clear differences in the physiological response trends between the hot water and tea conditions indicated that the tea ingredients had some effect on capillary blood flow. Notably, both hot water and tea were provided at the same temperature; therefore, the beverage temperature effects were not considered in this study. Previous studies report that brain activation changes depending on the complexity of calculations and anxiety levels, with differences in the left–right trend^52,53^. However, we observed no clear differences in the changes between the left and right sides in this study. Deoxygenated hemoglobin (deOxyHb) significantly decreased immediately after the start of the task, suggesting activation of brain function. Caffeine is known to constrict cerebral blood vessels and decrease cerebrate blood flow throughout the brain^54–56^, but the time to reach maximum blood concentration is 30–120 min after oral intake^57^. In previous studies, the caffeine intake ranged from 200 to 250 mg, approximately ten times higher than the caffeine intake in this study (green tea: 28 mg/roasted green tea: 26 mg). Regarding green tea ingredients such as catechins and theanine, one study reported a decrease in error rates and an improvement in simple reaction time in the Cognitrax Continuous Performance Test^58^. Theanine has been shown to directly affect the brain by increasing alpha waves without inducing drowsiness^59^, suggesting potential benefits such as improved working memory and relaxation^58,60^. However, the amounts of catechins (green tea: 94.4 mg/roasted green tea: 37.4 mg) and theanine (green tea: 6 mg/roasted green tea: not detected) ingested by participants in this study were considerably lower than those ingested by participants in previous studies (catechins 336.4 mg; theanine 97-200 mg). Other tea ingredients, such as the green tea polyphenol epigallocatechin-3-gallate (EGCG), have been shown to have physiologically neuroprotective effects^61^. EGCG at a low dose (135 mg) has been shown to decrease OxyHb and totalHb, with no change in deOxyHb^62^. Caffeine has been shown to decrease OxyHb; however, a previous study^63^ found that when caffeine was combined with l-theanine, the effect of caffeine on OxyHb disappeared, with no change in deOxyHb. Although we found similar results to those of previous studies, specific indicators related to acute cognitive performance due to tea components such as caffeine and theanine have not been definitively identified^64–66^. Considering the absorption time and intake amount, it is difficult to conclude that the ingestion of tea ingredients, such as caffeine and theanine, affected the capillary blood flow response in our study.

Other factors that can influence physiological indices over a short duration and in small quantities include aroma components. Aromatic compounds such as those found in plant-derived essential oils are known to have relaxation effects. For instance, orange essential oil has been reported to alleviate anxiety^67^, whereas rose oil is associated with an increased sense of happiness^68,69^. Igarashi et al.^70^ conducted psychological and physiological measurement experiments on female university students, exposing them to odor stimuli from rose and orange oils. Their results indicated that these odor stimuli induced an increase in psychological states such as “comfortable”, “relaxed”, and “natural” feelings, accompanied by a significant decrease in OxyHb concentration in the prefrontal cortex. Thus, olfactory stimuli such as aromatic oils have long been known to have mental relaxation effects. Regarding the aroma components of green tea, animal and human experiments have shown that green tea’s odor and linalool have anti-stress effects^71–74^. Furthermore, an experiment investigating the effects of odor stimuli from water and two types of green tea shows that the aroma of green tea increases feelings of relaxation and vitality following exposure to stress^15,16^. These results indicate that different types of green tea have varied effects on brain activity, task performance, and mood; the results of roasted green tea in this study support these conclusions. In addition, Höferl et al.^17^ examined autonomic nervous system activity (heart rate, blood pressure, and cutaneous electrical activity) through the inhalation of linalool, showing that the odorant relieved stress.

Ohata et al.^75^ investigated the effects of 2,3-dimethylylalazine (3DP), which has a nutty or cooked rice-like aroma, and 2,5-dimethyl-4-hydroxy-3(2H)-furanone (DMHF), known for its caramel-like and sweet flavor, on mood and physiological responses by the Maillard reaction. They reported that odor stimulations significantly increased miosis rate by the pupillary light reflex and fingertip temperature and significantly decreased OxyHb (for 2 min), especially in the frontal region. 3DP has the same basis as 2-ethyl-3,5-dimethylpyrazine, which is contained in the green tea and roasted green tea used in this study. Naturally, these are different chemical substances, but 2-ethyl-3,5-dimethylpyrazine is described as having an almond-like aroma; from an organic chemistry standpoint, it is highly likely that when inhaled, it will react similarly to 3DP. In addition to 3DP and DMHF, this study used Maillard reaction reagents and found similar physiological responses to all three chemicals, leading to the conclusion that odorant stimuli produced by the Maillard reaction can induce physiological relaxation by promoting mood and inhibiting sympathetic nerve activity. The Maillard reaction is a chemical reaction in which reducing sugars (carbohydrates) and amino compounds (proteins) are heated to generate brown substances such as melanoidin. In the production of roasted green tea, the Maillard reaction occurs during the roasting process, resulting in a brown color and distinctive aroma. Pyrazines are known to be components of the unique aroma of roasted green tea. Green tea also contains 2-ethyl-3,5-dimethylpyrazine, albeit to a lesser extent than roasted green tea. Therefore, we suggest that the physiological response in OxyHb observed in this study was due to a change in mood induced by aromatic stimulation of pyrazines produced by the Maillard reaction.

It has been reported that TBV responds to stress, correlates with the difficulty level of mental tasks^76,77^, and decreases during mental tasks compared with resting conditions. However, the physiological responses were extremely small in this study and hardly changed under the tea conditions. In the hot water condition, as in the other physiological responses, TBV started at a low level, likely associated with tension related to participation in the experiment, but increased during the repeated mental tasks, which is consistent with previous research. In contrast, under the tea condition, it is possible that the aromatic stimulation of the tea aroma caused peripheral vasoconstriction measured at the nose tip rather than a stress response to the mental arithmetic task. As Ohata et al. reported, if these odorant stimuli inhibited sympathetic nervous system activity and promoted both physiological and subjective relaxation, they might also be related to the unified response across multiple indicators of parasympathetic nervous system activity observed in this study. This study was limited to a number of young male participants. Moreover, considering the residual components of tea, it was not possible to counterbalance the order of water and tea, and to conduct with different tea conditions on the same day. Consequently, it was not possible to adequately confirm the changes associated with habituation to the experiment and the mental tasks, and directly compare the differences between types of tea. In the future, it will be necessary to expand the range of participants and explore a more robust experimental procedure or indicators to confirm the effects of habituation and types of tea. It is also desirable to investigate the effects of olfactory stimulation alone, rather than oral ingestion, on the aroma components suggested as factors for positive effects.

## Conclusion

This study comprehensively assessed various physiological responses, task performance, and subjective evaluations of the flavor and aroma of water, green tea, and roasted green tea (hojicha). Tea intake improved mental arithmetic task performance compared to the hot water condition. Furthermore, the results revealed tea consumption effects in promoting refreshment and facilitating subjective feelings of fatigue reduction and recovery. Through examining the multiple indicators, aromatic stimulation from tea drinks suggests the potential to exert positive physiological and subjective effects in a short duration and in small quantities, similar to amounts consumed daily. It is important to note that a limited number of young male participants and the effects of participation in physiological experiments and familiarity with tasks may have influenced the results. Further investigations are warranted to explore these aspects. It is also necessary to investigate the effects of aroma stimulation alone, without oral ingestion.

### Supplementary Information

Supplementary Tables.

## Data Availability

All data are maintained by the administrator in the university laboratory repository. The data sets generated during the current study are available from the corresponding author on reasonable request.
