# Author Correction: Effects of green tea and roasted green tea on human responses

**DOI:** 10.1038/s41598-024-61716-w

**Published:** 2024-05-16

**Authors:** Chie Kurosaka, Chika Tagata, Sae Nakagawa, Makoto Kobayashi, Shinji Miyake

**Affiliations:** 1 Department of Human, Information and Life Sciences, School of Health Sciences, University of Occupational and Environmental Health, Japan,, Kitakyushu, Fukuoka Japan; 2 Central Research Institute, ITOEN, Ltd, Makinohara, Shizuoka Japan; 3 Graduate School of Science and Technology, Chitose Institute of Science and Technology, Chitose, Hokkaido Japan

Correction to: *Scientific Reports* 10.1038/s41598-024-59383-y, published online 13 April 2024

The original version of this Article contained an error in Figure 3, where the labels for "Green Tea" and "Roasted Green Tea" were reversed. The original Figure 3 and accompanying legend appear below.

**Figure 3:**
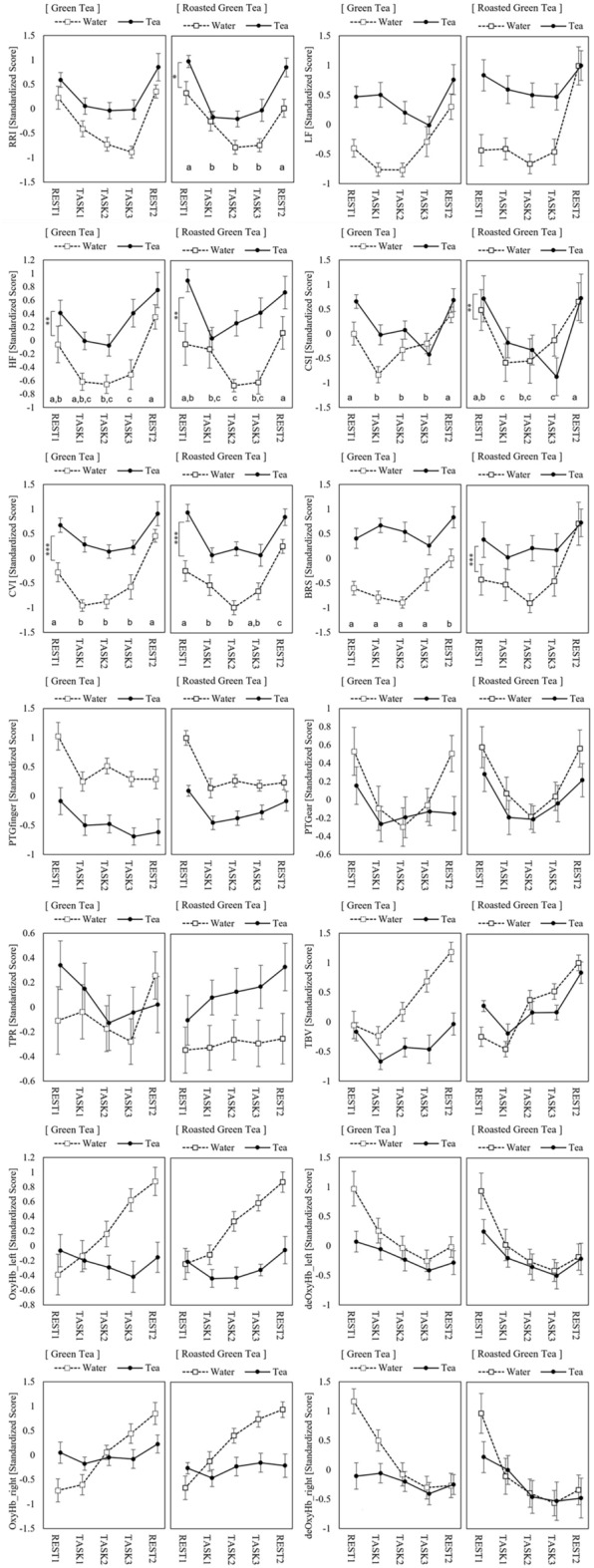
Physiological responses. Bars indicate standard errors of the mean. The alphabets (**a**–**d**) indicate homogenous subset results based on the REGW F-test. Asterisks indicate the significant differences between sessions (***p < 0.001, **p < 0.01).

The original Article has been corrected.

