# Supplementary material for: Effects of green tea and roasted green tea on human responses
Source: Sci Rep. 2024 Apr 13;14:8588. doi: 10.1038/s41598-024-59383-y (PMC11016062; doi:10.1038/s41598-024-59383-y)
Supplement: Supplementary file 1 — Supplementary Tables. [file 41598_2024_59383_MOESM1_ESM.docx]

Supplementary Table 1. Results of the component analysis of the beverages. Theanine, caffeine anhydrous, and catechins are measured per 100g, and pyrazines are measured per 1L. A “<” in the table indicates that the content is less than the quantitative limit.

|  | Water | Green tea | Roasted green tea | Determination limit |
| --- | --- | --- | --- | --- |
| Theanine | Not deleted | 3.0mg | Not deleted | 1.0mg |
| Caffeine anhydrous | Not deleted | 14.0mg | 13.0mg | 1.0mg |
| Catechin | Not deleted | 3.0mg | 1.8mg | 0.5mg |
| Epicatechin | Not deleted | 1.7mg | 0.9mg | 0.5mg |
| Gallocatechin | Not deleted | 12.0mg | 5.2mg | 0.5mg |
| Epigallocatechin | Not deleted | 6.6mg | 2.6mg | 0.5mg |
| Epigallocatechin gallate | Not deleted | 10mg | 3.2mg | 0.5mg |
| Gallocatechin gallate | Not deleted | 10mg | 3.3mg | 0.5mg |
| Epicatechin gallate | Not deleted | 2.0mg | 0.8mg | 0.5mg |
| Catechin gallate | Not deleted | 1.9mg | 0.9mg | 0.5mg |
| 2-Ethyl-3,5-dimethylpyrazine ^*)^ | ＜1.0μg | 6.9μg | 29.0μg |  |
| Tetramethylpyrazine | ＜0.5μg | ＜0.5μg | 1.2μg |  |
| 2,3-Diethyl-5-methylpyrazine | ＜1.0μg | ＜1.0μg | 1.2μg |  |

**Note*: In green tea and roasted green tea, the 2,3-diethyl-5-methylpyrazine peak overlapped with other peaks (isomers and presumed isomers). The overlapping peaks were subtracted from the quantification. In the case of water, a small amount of a peak that appeared to be derived from other ingredients was detected at the peak detection time of 2,3-diethyl-5-methylpyrazine; therefore, m/z 150 was used instead of m/z 121 for quantification.

**Supplementary Table 2.** Results of the repeated two-way ANOVA in the task performance and subjective assessments. Asterisks indicate the main effects and interactions for each factor (*** p<0.001, ** p<0.01, *p<0.05, † p<0.10).

(1) Water and Green Tea condition

|  |  |  | *df* | *F value* | *ε* | *1-β* | *η^2^* | *p* |  |
| --- | --- | --- | --- | --- | --- | --- | --- | --- | --- |
| Task  Performance | Response Rate | Session | 1.00 | 3.40 | 1.00 | 0.41 | 0.18 | 0.084 | † |
|  |  | Block | 1.62 | 0.60 | 0.81 | 0.13 | 0.04 | 0.521 |  |
|  |  | Session×Block | 1.64 | 3.43 | 0.82 | 0.54 | 0.18 | 0.056 | † |
|  | Correct Rate | Session | 1.00 | 7.09 | 1.00 | 0.71 | 0.31 | 0.017 | * |
|  |  | Block | 1.61 | 1.93 | 0.80 | 0.33 | 0.11 | 0.171 |  |
|  |  | Session×Block | 1.30 | 1.75 | 0.65 | 0.27 | 0.10 | 0.202 |  |
|  | Mean Level | Session | 1.00 | 9.95 | 1.00 | 0.84 | 0.38 | 0.006 | ** |
|  |  | Block | 1.94 | 0.40 | 0.97 | 0.11 | 0.02 | 0.670 |  |
|  |  | Session×Block | 1.62 | 2.87 | 0.81 | 0.47 | 0.15 | 0.084 | † |
| NASA-TLX | FR | Session | 1.00 | 0.16 | 1.00 | 0.07 | 0.01 | 0.696 |  |
|  |  | Block | 1.70 | 0.40 | 0.85 | 0.10 | 0.02 | 0.638 |  |
|  |  | Session×Block | 1.71 | 1.24 | 0.86 | 0.23 | 0.07 | 0.300 |  |
|  | OP | Session | 1.00 | 0.01 | 1.00 | 0.05 | 0.00 | 0.907 |  |
|  |  | Block | 1.85 | 0.22 | 0.93 | 0.08 | 0.01 | 0.785 |  |
|  |  | Session×Block | 1.71 | 0.79 | 0.85 | 0.16 | 0.05 | 0.447 |  |
|  | EF | Session | 1.00 | 0.01 | 1.00 | 0.05 | 0.00 | 0.907 |  |
|  |  | Block | 1.85 | 0.22 | 0.93 | 0.08 | 0.01 | 0.785 |  |
|  |  | Session×Block | 1.71 | 0.79 | 0.85 | 0.16 | 0.05 | 0.447 |  |
|  | TD | Session | 1.00 | 2.36 | 1.00 | 0.30 | 0.13 | 0.144 |  |
|  |  | Block | 1.79 | 2.88 | 0.90 | 0.49 | 0.15 | 0.078 | † |
|  |  | Session×Block | 1.80 | 5.23 | 0.90 | 0.76 | 0.25 | 0.014 | * |
|  | PD | Session | 1.00 | 7.07 | 1.00 | 0.70 | 0.31 | 0.017 | * |
|  |  | Block | 1.64 | 0.97 | 0.82 | 0.19 | 0.06 | 0.375 |  |
|  |  | Session×Block | 1.92 | 0.02 | 0.96 | 0.05 | 0.00 | 0.973 |  |
|  | MD | Session | 1.00 | 0.81 | 1.00 | 0.14 | 0.05 | 0.381 |  |
|  |  | Block | 1.78 | 0.58 | 0.89 | 0.13 | 0.04 | 0.545 |  |
|  |  | Session×Block | 1.54 | 3.70 | 0.77 | 0.56 | 0.19 | 0.050 | * |
|  | AWWL | Session | 1.00 | 1.42 | 1.00 | 0.20 | 0.08 | 0.250 |  |
|  |  | Block | 1.64 | 0.52 | 0.82 | 0.12 | 0.03 | 0.567 |  |
|  |  | Session×Block | 1.59 | 4.60 | 0.80 | 0.66 | 0.22 | 0.026 | * |
| FLOW | AIM | Session | 1.00 | 0.70 | 1.00 | 0.12 | 0.04 | 0.414 |  |
|  |  | Block | 1.50 | 0.92 | 0.75 | 0.17 | 0.05 | 0.384 |  |
|  |  | Session×Block | 1.28 | 0.66 | 0.64 | 0.13 | 0.04 | 0.459 |  |
|  | FEEDBACK | Session | 1.00 | 7.24 | 1.00 | 0.72 | 0.29 | 0.015 | * |
|  |  | Block | 1.41 | 0.39 | 0.70 | 0.10 | 0.02 | 0.610 |  |
|  |  | Session×Block | 1.40 | 2.18 | 0.70 | 0.34 | 0.11 | 0.146 |  |
|  | CHALLENGE | Session | 1.00 | 5.58 | 1.00 | 0.61 | 0.24 | 0.030 | * |
|  |  | Block | 1.63 | 2.06 | 0.82 | 0.35 | 0.10 | 0.152 |  |
|  |  | Session×Block | 1.68 | 0.46 | 0.84 | 0.11 | 0.03 | 0.601 |  |
|  | TIME | Session | 1.00 | 0.63 | 1.00 | 0.12 | 0.03 | 0.438 |  |
|  |  | Block | 1.94 | 3.00 | 0.97 | 0.54 | 0.14 | 0.064 | † |
|  |  | Session×Block | 1.81 | 2.84 | 0.91 | 0.49 | 0.14 | 0.078 | † |
|  | FLOW | Session | 1.00 | 4.14 | 1.00 | 0.49 | 0.19 | 0.057 | † |
|  |  | Block | 1.37 | 2.38 | 0.68 | 0.36 | 0.12 | 0.128 |  |
|  |  | Session×Block | 1.46 | 0.76 | 0.73 | 0.15 | 0.04 | 0.437 |  |
|  | DJR | Session | 1.00 | 0.37 | 1.00 | 0.09 | 0.02 | 0.549 |  |
|  |  | Block | 1.88 | 2.39 | 0.94 | 0.44 | 0.11 | 0.109 |  |
|  |  | Session×Block | 1.88 | 2.64 | 0.94 | 0.48 | 0.12 | 0.088 | † |

**Note:* FR: frustration level, OP: own performance, EF: effort, TD; temporal demand, PD: physical demand, MD: mental demand, AWWL: adaptive weighted workload, DJR: duration judgment ratio; FLOW: Flow Experience Checklist.

(2) Water and Roasted Green Tea condition.

|  |  | | *df* | *F value* | *ε* | *1-β* | *η^2^* | *p* |  |
| --- | --- | --- | --- | --- | --- | --- | --- | --- | --- |
| Task  Performance | Response Rate | Session | 1.00 | 4.55 | 1.00 | 0.52 | 0.22 | 0.049 | * |
|  |  | Block | 1.92 | 8.93 | 0.96 | 0.95 | 0.36 | 0.001 | *** |
|  |  | Session×Block | 1.68 | 1.02 | 0.84 | 0.20 | 0.06 | 0.363 |  |
|  | Correct Rate | Session | 1.00 | 8.99 | 1.00 | 0.80 | 0.36 | 0.008 | ** |
|  |  | Block | 1.85 | 5.41 | 0.92 | 0.78 | 0.25 | 0.011 | * |
|  |  | Session×Block | 1.84 | 0.37 | 0.92 | 0.10 | 0.02 | 0.673 |  |
|  | Mean Level | Session | 1.00 | 3.76 | 1.00 | 0.44 | 0.19 | 0.071 | † |
|  |  | Block | 1.75 | 4.19 | 0.88 | 0.65 | 0.21 | 0.030 | * |
|  |  | Session×Block | 1.70 | 1.33 | 0.85 | 0.25 | 0.08 | 0.277 |  |
| NASA-TLX | FR | Session | 1.00 | 0.35 | 1.00 | 0.09 | 0.02 | 0.561 |  |
|  |  | Block | 1.56 | 0.23 | 0.78 | 0.08 | 0.01 | 0.739 |  |
|  |  | Session×Block | 1.94 | 2.15 | 0.97 | 0.40 | 0.12 | 0.135 |  |
|  | OP | Session | 1.00 | 0.37 | 1.00 | 0.09 | 0.02 | 0.550 |  |
|  |  | Block | 1.96 | 1.12 | 0.98 | 0.23 | 0.07 | 0.339 |  |
|  |  | Session×Block | 1.90 | 0.39 | 0.95 | 0.11 | 0.02 | 0.668 |  |
|  | EF | Session | 1.00 | 0.37 | 1.00 | 0.09 | 0.02 | 0.550 |  |
|  |  | Block | 1.96 | 1.12 | 0.98 | 0.23 | 0.07 | 0.339 |  |
|  |  | Session×Block | 1.90 | 0.39 | 0.95 | 0.11 | 0.02 | 0.668 |  |
|  | TD | Session | 1.00 | 2.90 | 1.00 | 0.36 | 0.15 | 0.108 |  |
|  |  | Block | 1.61 | 6.46 | 0.81 | 0.82 | 0.29 | 0.008 | ** |
|  |  | Session×Block | 1.97 | 0.31 | 0.98 | 0.09 | 0.02 | 0.732 |  |
|  | PD | Session | 1.00 | 0.38 | 1.00 | 0.09 | 0.02 | 0.544 |  |
|  |  | Block | 1.71 | 0.46 | 0.86 | 0.11 | 0.03 | 0.606 |  |
|  |  | Session×Block | 1.96 | 4.23 | 0.98 | 0.69 | 0.21 | 0.024 | * |
|  | MD | Session | 1.00 | 1.10 | 1.00 | 0.17 | 0.06 | 0.310 |  |
|  |  | Block | 1.56 | 0.10 | 0.78 | 0.06 | 0.01 | 0.856 |  |
|  |  | Session×Block | 1.72 | 1.87 | 0.86 | 0.33 | 0.10 | 0.177 |  |
|  | AWWL | Session | 1.00 | 0.25 | 1.00 | 0.08 | 0.02 | 0.625 |  |
|  |  | Block | 1.45 | 2.14 | 0.73 | 0.34 | 0.12 | 0.151 |  |
|  |  | Session×Block | 1.97 | 0.87 | 0.99 | 0.19 | 0.05 | 0.427 |  |
| FLOW | AIM | Session | 1.00 | 2.68 | 1.00 | 0.34 | 0.13 | 0.119 |  |
|  |  | Block | 1.89 | 0.35 | 0.94 | 0.10 | 0.02 | 0.693 |  |
|  |  | Session×Block | 1.87 | 0.50 | 0.94 | 0.12 | 0.03 | 0.600 |  |
|  | FEEDBACK | Session | 1.00 | 0.02 | 1.00 | 0.05 | 0.00 | 0.886 |  |
|  |  | Block | 1.63 | 0.36 | 0.81 | 0.10 | 0.02 | 0.657 |  |
|  |  | Session×Block | 1.70 | 0.56 | 0.85 | 0.13 | 0.03 | 0.550 |  |
|  | CHALLENGE | Session | 1.00 | 0.56 | 1.00 | 0.11 | 0.03 | 0.465 |  |
|  |  | Block | 1.74 | 3.88 | 0.87 | 0.62 | 0.18 | 0.037 | * |
|  |  | Session×Block | 1.92 | 0.09 | 0.96 | 0.06 | 0.00 | 0.908 |  |
|  | TIME | Session | 1.00 | 0.10 | 1.00 | 0.06 | 0.01 | 0.759 |  |
|  |  | Block | 1.83 | 0.50 | 0.92 | 0.12 | 0.03 | 0.597 |  |
|  |  | Session×Block | 1.96 | 0.20 | 0.98 | 0.08 | 0.01 | 0.815 |  |
|  | FLOW | Session | 1.00 | 0.08 | 1.00 | 0.06 | 0.00 | 0.787 |  |
|  |  | Block | 1.58 | 1.07 | 0.79 | 0.20 | 0.06 | 0.341 |  |
|  |  | Session×Block | 1.93 | 0.07 | 0.96 | 0.06 | 0.00 | 0.925 |  |
|  | DJR | Session | 1.00 | 0.69 | 1.00 | 0.12 | 0.04 | 0.415 |  |
|  |  | Block | 1.79 | 2.42 | 0.90 | 0.43 | 0.11 | 0.110 |  |
|  |  | Session×Block | 1.76 | 0.89 | 0.88 | 0.18 | 0.04 | 0.410 |  |

**Note:* FR: frustration level, OP: own performance, EF: effort, TD; temporal demand, PD: physical demand, MD: mental demand, AWWL: adaptive weighted workload, DJR: duration judgment ratio; FLOW: Flow experience checklist.

**Supplementary Table 3.** Results of the repeated two-way ANOVA for the physiological responses. Asterisks indicate the main effects and interactions for each factor (*** p<0.001, ** p<0.01, *p<0.05, † p<0.10).

(1) Water and Green Tea condition

|  |  |  | *df* | *F value* | *ε* | *1-β* | *η^2^* | *p* |  |
| --- | --- | --- | --- | --- | --- | --- | --- | --- | --- |
| HRV | RRI | Session | 1.00 | 7.93 | 1.00 | 0.76 | 0.29 | 0.011 | * |
|  |  | Block | 2.93 | 13.47 | 0.73 | 1.00 | 0.41 | 0.000 | *** |
|  |  | Session×Block | 3.55 | 3.23 | 0.89 | 0.77 | 0.15 | 0.021 | * |
|  | LF | Session | 1.00 | 15.17 | 1.00 | 0.96 | 0.46 | 0.001 | ** |
|  |  | Block | 3.11 | 5.18 | 0.78 | 0.91 | 0.22 | 0.003 | ** |
|  |  | Session×Block | 3.45 | 3.58 | 0.86 | 0.81 | 0.17 | 0.015 | * |
|  | HF | Session | 1.00 | 12.61 | 1.00 | 0.92 | 0.41 | 0.002 | ** |
|  |  | Block | 2.87 | 2.97 | 0.72 | 0.66 | 0.14 | 0.042 | * |
|  |  | Session×Block | 3.61 | 2.13 | 0.90 | 0.57 | 0.11 | 0.093 | † |
|  | LF/HF | Session | 1.00 | 0.51 | 1.00 | 0.10 | 0.03 | 0.485 |  |
|  |  | Block | 2.62 | 1.26 | 0.66 | 0.30 | 0.07 | 0.298 |  |
|  |  | Session×Block | 3.41 | 1.83 | 0.85 | 0.48 | 0.09 | 0.145 |  |
|  | SD1 | Session | 1.00 | 16.26 | 1.00 | 0.97 | 0.47 | 0.001 | *** |
|  |  | Block | 2.98 | 3.20 | 0.75 | 0.71 | 0.15 | 0.031 | * |
|  |  | Session×Block | 3.42 | 2.08 | 0.85 | 0.54 | 0.10 | 0.104 |  |
|  | SD2 | Session | 1.00 | 24.24 | 1.00 | 1.00 | 0.57 | 0.000 | *** |
|  |  | Block | 3.38 | 10.60 | 0.85 | 1.00 | 0.37 | 0.000 | *** |
|  |  | Session×Block | 3.64 | 1.63 | 0.91 | 0.45 | 0.08 | 0.182 |  |
|  | CSI | Session | 1.00 | 0.01 | 1.00 | 0.05 | 0.00 | 0.924 |  |
|  |  | Block | 3.37 | 3.95 | 0.84 | 0.84 | 0.18 | 0.010 | ** |
|  |  | Session×Block | 3.39 | 0.80 | 0.85 | 0.23 | 0.04 | 0.510 |  |
|  | CVI | Session | 1.00 | 30.72 | 1.00 | 1.00 | 0.63 | 0.000 | *** |
|  |  | Block | 3.58 | 9.35 | 0.90 | 1.00 | 0.34 | 0.000 | *** |
|  |  | Session×Block | 3.33 | 1.97 | 0.83 | 0.51 | 0.10 | 0.122 |  |
| Blood  Pressure | SBP | Session | 1.00 | 0.06 | 1.00 | 0.06 | 0.00 | 0.803 |  |
|  |  | Block | 3.12 | 7.20 | 0.78 | 0.98 | 0.29 | 0.000 | *** |
|  |  | Session×Block | 2.96 | 0.78 | 0.74 | 0.21 | 0.04 | 0.506 |  |
|  | DBP | Session | 1.00 | 0.04 | 1.00 | 0.05 | 0.00 | 0.846 |  |
|  |  | Block | 2.41 | 2.94 | 0.60 | 0.59 | 0.14 | 0.054 | † |
|  |  | Session×Block | 2.40 | 0.88 | 0.60 | 0.21 | 0.05 | 0.438 |  |
|  | MBP | Session | 1.00 | 0.00 | 1.00 | 0.05 | 0.00 | 0.967 |  |
|  |  | Block | 2.60 | 3.71 | 0.65 | 0.73 | 0.17 | 0.022 | * |
|  |  | Session×Block | 2.45 | 0.96 | 0.61 | 0.22 | 0.05 | 0.407 |  |
|  | BRS | Session | 1.00 | 2.53 | 1.00 | 0.33 | 0.12 | 0.129 |  |
|  |  | Block | 2.70 | 4.43 | 0.68 | 0.82 | 0.20 | 0.010 | ** |
|  |  | Session×Block | 3.28 | 1.26 | 0.82 | 0.33 | 0.07 | 0.298 |  |
| Hemodaynamics | CO | Session | 1.00 | 7.82 | 1.00 | 0.75 | 0.30 | 0.012 | * |
|  |  | Block | 3.22 | 5.62 | 0.80 | 0.94 | 0.24 | 0.002 | ** |
|  |  | Session×Block | 3.34 | 0.23 | 0.84 | 0.09 | 0.01 | 0.896 |  |
|  | TPR | Session | 1.00 | 2.43 | 1.00 | 0.31 | 0.12 | 0.137 |  |
|  |  | Block | 3.06 | 0.86 | 0.77 | 0.23 | 0.05 | 0.468 |  |
|  |  | Session×Block | 2.94 | 0.42 | 0.73 | 0.13 | 0.02 | 0.738 |  |
| Plethysmogram | PTGear | Session | 1.00 | 1.41 | 1.00 | 0.20 | 0.07 | 0.249 |  |
|  |  | Block | 2.94 | 3.70 | 0.74 | 0.77 | 0.16 | 0.017 | * |
|  |  | Session×Block | 3.04 | 0.45 | 0.76 | 0.14 | 0.02 | 0.721 |  |
|  | PTGfinger | Session | 1.00 | 15.42 | 1.00 | 0.96 | 0.45 | 0.001 | *** |
|  |  | Block | 3.24 | 8.36 | 0.81 | 0.99 | 0.31 | 0.000 | *** |
|  |  | Session×Block | 2.86 | 3.16 | 0.71 | 0.69 | 0.14 | 0.034 | * |
| Peripheral Blood Flow | TBV | Session | 1.00 | 0.00 | 1.00 | 0.05 | 0.00 | 0.958 |  |
|  |  | Block | 2.95 | 24.49 | 0.74 | 1.00 | 0.56 | 0.000 | *** |
|  |  | Session×Block | 3.06 | 4.06 | 0.77 | 0.82 | 0.18 | 0.010 | * |
|  | TBF | Session | 1.00 | 0.06 | 1.00 | 0.06 | 0.00 | 0.809 |  |
|  |  | Block | 2.57 | 21.52 | 0.64 | 1.00 | 0.53 | 0.000 | *** |
|  |  | Session×Block | 2.43 | 5.26 | 0.61 | 0.86 | 0.22 | 0.006 | ** |
| Electrodermal Activity | SPL | Session | 1.00 | 3.90 | 1.00 | 0.47 | 0.17 | 0.063 | † |
|  |  | Block | 2.77 | 2.00 | 0.69 | 0.47 | 0.10 | 0.129 |  |
|  |  | Session×Block | 2.70 | 1.03 | 0.67 | 0.25 | 0.05 | 0.381 |  |
| NIRS | deOxyHb_left | Session | 1.00 | 0.48 | 1.00 | 0.10 | 0.02 | 0.498 |  |
|  |  | Block | 2.51 | 8.15 | 0.63 | 0.97 | 0.30 | 0.000 | *** |
|  |  | Session×Block | 2.06 | 1.47 | 0.51 | 0.30 | 0.07 | 0.242 |  |
|  | OxyHb_left | Session | 1.00 | 13.15 | 1.00 | 0.93 | 0.41 | 0.002 | ** |
|  |  | Block | 2.26 | 7.90 | 0.56 | 0.96 | 0.29 | 0.001 | *** |
|  |  | Session×Block | 2.54 | 5.45 | 0.64 | 0.88 | 0.22 | 0.004 | ** |
|  | totalHb_lfet | Session | 1.00 | 36.86 | 1.00 | 1.00 | 0.66 | 0.000 | *** |
|  |  | Block | 2.29 | 4.60 | 0.57 | 0.79 | 0.19 | 0.012 | * |
|  |  | Session×Block | 3.02 | 1.89 | 0.76 | 0.47 | 0.09 | 0.141 |  |
|  | deOxyHb_right | Session | 1.00 | 0.18 | 1.00 | 0.07 | 0.01 | 0.680 |  |
|  |  | Block | 2.89 | 6.18 | 0.72 | 0.95 | 0.25 | 0.001 | ** |
|  |  | Session×Block | 2.47 | 1.46 | 0.62 | 0.33 | 0.07 | 0.241 |  |
|  | OxyHb_right | Session | 1.00 | 4.89 | 1.00 | 0.56 | 0.20 | 0.039 | * |
|  |  | Block | 2.55 | 7.36 | 0.64 | 0.96 | 0.28 | 0.001 | *** |
|  |  | Session×Block | 2.49 | 11.32 | 0.62 | 1.00 | 0.37 | 0.000 | *** |
|  | totalHb_right | Session | 1.00 | 2.58 | 1.00 | 0.33 | 0.12 | 0.125 |  |
|  |  | Block | 2.61 | 4.25 | 0.65 | 0.79 | 0.18 | 0.013 | * |
|  |  | Session×Block | 2.47 | 6.05 | 0.62 | 0.91 | 0.24 | 0.003 | ** |

**Note:* RRI: R wave interval; LF and HF: low/high components of heart rate variability; SD1, SD2, CSI, and CVI: Poincaré plot indices; SBP, DBP, and MBP: systolic/diastolic/mean blood pressure; BRS: baroreflex sensitivity; CO: cardiac output; TPR: total peripheral vascular resistance; PTG: plethysmogram on finger/ear; TBV and TBF: tissue blood volume/flow; SPL: skin potential level; (de)OxyHb: oxygen hemoglobin.

(2) Water and Roasted Green Tea condition

|  |  | |  | | *df* | | *F value* | | *ε* | | *1-β* | | *η^2^* | | *p* |  |
| --- | --- | --- | --- | --- | --- | --- | --- | --- | --- | --- | --- | --- | --- | --- | --- | --- |
| HRV | RRI | | Session | | 1.00 | | 6.20 | | 1.00 | | 0.66 | | 0.25 | | 0.022 | * |
|  |  | | Block | | 2.25 | | 14.03 | | 0.56 | | 1.00 | | 0.42 | | 0.000 | *** |
|  |  | | Session×Block | | 3.13 | | 1.25 | | 0.78 | | 0.32 | | 0.06 | | 0.301 |  |
|  | LF | | Session | | 1.00 | | 23.33 | | 1.00 | | 1.00 | | 0.56 | | 0.000 | *** |
|  |  | | Block | | 2.77 | | 4.48 | | 0.69 | | 0.83 | | 0.20 | | 0.009 | ** |
|  |  | | Session×Block | | 3.18 | | 2.81 | | 0.79 | | 0.66 | | 0.13 | | 0.045 | * |
|  | HF | | Session | | 1.00 | | 10.46 | | 1.00 | | 0.86 | | 0.37 | | 0.005 | ** |
|  |  | | Block | | 2.79 | | 6.57 | | 0.70 | | 0.95 | | 0.27 | | 0.001 | ** |
|  |  | | Session×Block | | 3.08 | | 0.62 | | 0.77 | | 0.17 | | 0.03 | | 0.607 |  |
|  | LFHF | | Session | | 1.00 | | 5.29 | | 1.00 | | 0.59 | | 0.23 | | 0.034 | * |
|  |  | | Block | | 3.20 | | 0.83 | | 0.80 | | 0.22 | | 0.04 | | 0.491 |  |
|  |  | | Session×Block | | 3.41 | | 1.92 | | 0.85 | | 0.50 | | 0.10 | | 0.129 |  |
|  | SD1 | | Session | | 1.00 | | 16.62 | | 1.00 | | 0.97 | | 0.48 | | 0.001 | *** |
|  |  | | Block | | 2.67 | | 4.51 | | 0.67 | | 0.83 | | 0.20 | | 0.009 | ** |
|  |  | | Session×Block | | 2.63 | | 0.88 | | 0.66 | | 0.21 | | 0.05 | | 0.447 |  |
|  | SD2 | | Session | | 1.00 | | 38.09 | | 1.00 | | 1.00 | | 0.68 | | 0.000 | *** |
|  |  | | Block | | 3.46 | | 14.85 | | 0.86 | | 1.00 | | 0.45 | | 0.000 | *** |
|  |  | | Session×Block | | 3.03 | | 2.03 | | 0.76 | | 0.49 | | 0.10 | | 0.121 |  |
|  | CSI | | Session | | 1.00 | | 8.49 | | 1.00 | | 0.79 | | 0.32 | | 0.009 | ** |
|  |  | | Block | | 3.64 | | 7.65 | | 0.91 | | 0.99 | | 0.30 | | 0.000 | *** |
|  |  | | Session×Block | | 3.38 | | 1.98 | | 0.85 | | 0.52 | | 0.10 | | 0.119 |  |
|  | CVI | | Session | | 1.00 | | 29.39 | | 1.00 | | 1.00 | | 0.62 | | 0.000 | *** |
|  |  | | Block | | 3.14 | | 13.09 | | 0.78 | | 1.00 | | 0.42 | | 0.000 | *** |
|  |  | | Session×Block | | 3.01 | | 1.63 | | 0.75 | | 0.40 | | 0.08 | | 0.193 |  |
| Blood  Pressure | SBP | | Session | | 1.00 | | 0.23 | | 1.00 | | 0.07 | | 0.01 | | 0.638 |  |
|  |  | | Block | | 3.14 | | 3.85 | | 0.78 | | 0.81 | | 0.17 | | 0.013 | * |
|  |  | | Session×Block | | 3.24 | | 1.90 | | 0.81 | | 0.49 | | 0.09 | | 0.135 |  |
|  | DBP | | Session | | 1.00 | | 0.56 | | 1.00 | | 0.11 | | 0.03 | | 0.462 |  |
|  |  | | Block | | 2.98 | | 0.93 | | 0.74 | | 0.24 | | 0.05 | | 0.432 |  |
|  |  | | Session×Block | | 3.08 | | 0.81 | | 0.77 | | 0.22 | | 0.04 | | 0.495 |  |
|  | MBP | | Session | | 1.00 | | 0.37 | | 1.00 | | 0.09 | | 0.02 | | 0.548 |  |
|  |  | | Block | | 2.98 | | 1.58 | | 0.75 | | 0.39 | | 0.08 | | 0.206 |  |
|  |  | | Session×Block | | 3.14 | | 0.85 | | 0.78 | | 0.23 | | 0.04 | | 0.478 |  |
|  | BRS | Session | | 1.00 | | 31.21 | | 1.00 | | 1.00 | | 0.65 | | 0.000 | | *** |
|  |  | Block | | 3.74 | | 3.65 | | 0.93 | | 0.84 | | 0.18 | | 0.011 | | * |
|  |  | Session×Block | | 3.37 | | 2.51 | | 0.84 | | 0.63 | | 0.13 | | 0.061 | | † |
| Hemodaynamics | CO | | Session | | 1.00 | | 3.00 | | 1.00 | | 0.38 | | 0.14 | | 0.099 | † |
|  |  | | Block | | 2.11 | | 8.45 | | 0.53 | | 0.96 | | 0.31 | | 0.001 | *** |
|  |  | | Session×Block | | 3.10 | | 0.89 | | 0.77 | | 0.24 | | 0.04 | | 0.455 |  |
|  | TPR | | Session | | 1.00 | | 0.23 | | 1.00 | | 0.07 | | 0.01 | | 0.639 |  |
|  |  | | Block | | 2.75 | | 1.32 | | 0.69 | | 0.32 | | 0.06 | | 0.279 |  |
|  |  | | Session×Block | | 2.85 | | 1.07 | | 0.71 | | 0.27 | | 0.05 | | 0.367 |  |
| Plethysmogram | PTGear | | Session | | 1.00 | | 1.11 | | 1.00 | | 0.17 | | 0.06 | | 0.304 |  |
|  |  | | Block | | 3.19 | | 3.70 | | 0.80 | | 0.80 | | 0.16 | | 0.015 | * |
|  |  | | Session×Block | | 3.46 | | 1.00 | | 0.87 | | 0.28 | | 0.05 | | 0.408 |  |
|  | PTGfinger | | Session | | 1.00 | | 36.88 | | 1.00 | | 1.00 | | 0.66 | | 0.000 | *** |
|  |  | | Block | | 2.91 | | 3.84 | | 0.73 | | 0.78 | | 0.17 | | 0.015 | * |
|  |  | | Session×Block | | 2.54 | | 0.34 | | 0.63 | | 0.11 | | 0.02 | | 0.761 |  |
| Peripheral Blood Flow | TBV | | Session | | 1.00 | | 11.50 | | 1.00 | | 0.90 | | 0.38 | | 0.003 | ** |
|  |  | | Block | | 2.25 | | 9.54 | | 0.56 | | 0.98 | | 0.33 | | 0.000 | *** |
|  |  | | Session×Block | | 3.26 | | 4.68 | | 0.82 | | 0.89 | | 0.20 | | 0.004 | ** |
|  | TBF | | Session | | 1.00 | | 6.87 | | 1.00 | | 0.70 | | 0.27 | | 0.017 | * |
|  |  | | Block | | 2.31 | | 8.45 | | 0.58 | | 0.97 | | 0.31 | | 0.000 | *** |
|  |  | | Session×Block | | 3.14 | | 4.75 | | 0.79 | | 0.89 | | 0.20 | | 0.004 | ** |
| Electrodermal Activity | SPL | | Session | | 1.00 | | 0.24 | | 1.00 | | 0.08 | | 0.01 | | 0.628 |  |
|  |  | | Block | | 2.42 | | 2.30 | | 0.60 | | 0.49 | | 0.11 | | 0.102 |  |
|  |  | | Session×Block | | 2.71 | | 0.97 | | 0.68 | | 0.24 | | 0.05 | | 0.406 |  |
| NIRS | deOxyHb_left | | Session | | 1.00 | | 1.99 | | 1.00 | | 0.27 | | 0.09 | | 0.174 |  |
|  |  | | Block | | 2.32 | | 7.70 | | 0.58 | | 0.96 | | 0.29 | | 0.001 | *** |
|  |  | | Session×Block | | 1.66 | | 1.55 | | 0.41 | | 0.28 | | 0.08 | | 0.230 |  |
|  | OxyHb_left | | Session | | 1.00 | | 5.16 | | 1.00 | | 0.58 | | 0.21 | | 0.035 | * |
|  |  | | Block | | 2.26 | | 2.78 | | 0.57 | | 0.55 | | 0.13 | | 0.067 | † |
|  |  | | Session×Block | | 2.79 | | 5.77 | | 0.70 | | 0.92 | | 0.23 | | 0.002 | ** |
|  | totalHb_lfet | | Session | | 1.00 | | 5.39 | | 1.00 | | 0.60 | | 0.22 | | 0.031 | * |
|  |  | | Block | | 2.11 | | 1.14 | | 0.53 | | 0.24 | | 0.06 | | 0.333 |  |
|  |  | | Session×Block | | 2.88 | | 1.15 | | 0.72 | | 0.29 | | 0.06 | | 0.337 |  |
|  | deOxyHb_right | | Session | | 1.00 | | 3.07 | | 1.00 | | 0.38 | | 0.14 | | 0.096 | † |
|  |  | | Block | | 2.34 | | 7.66 | | 0.58 | | 0.96 | | 0.29 | | 0.001 | *** |
|  |  | | Session×Block | | 2.59 | | 6.40 | | 0.65 | | 0.93 | | 0.25 | | 0.002 | ** |
|  | OxyHb_right | | Session | | 1.00 | | 0.00 | | 1.00 | | 0.05 | | 0.00 | | 0.968 |  |
|  |  | | Block | | 2.17 | | 8.46 | | 0.54 | | 0.96 | | 0.31 | | 0.001 | *** |
|  |  | | Session×Block | | 2.76 | | 6.56 | | 0.69 | | 0.95 | | 0.26 | | 0.001 | ** |
|  | totalHb_right | | Session | | 1.00 | | 3.13 | | 1.00 | | 0.39 | | 0.14 | | 0.093 | † |
|  |  | | Block | | 2.39 | | 2.76 | | 0.60 | | 0.56 | | 0.13 | | 0.065 | † |
|  |  | | Session×Block | | 2.95 | | 1.57 | | 0.74 | | 0.39 | | 0.08 | | 0.206 |  |

**Note:* RRI: R wave interval; LF and HF: low/high components of heart rate variability; SD1, SD2, CSI, and CVI: Poincaré plot indices; SBP, DBP, and MBP: systolic/diastolic/mean blood pressure; BRS: baroreflex sensitivity; CO: cardiac output; TPR: total peripheral vascular resistance; PTG: plethysmogram on finger/ear; TBV and TBF: tissue blood volume/flow; SPL: skin potential level; (de)OxyHb: oxygen hemoglobin.
